# Poly(Alkoxythiophene) Rod‐Coil Block Copolymers for Organic Electrochemical Transistors

**DOI:** 10.1002/adma.74978

**Published:** 2026-09-13

**Authors:** Junfu Tian, Wentao Shan, Rachael Found, Paola Mantegazza, Francesco Leoni, Andreas W. Erhardt, Yuyun Yao, Matus Stredansky, Arianna Magni, Adam Marks, Elena Chulanova, Zeinab Hamid, Yueying Huang, Wanpeng Lu, Xian'e Li, Baopu Zhang, Qingpei Wan, Christina Kousseff, Matilde Concilio, Ruth Theresia Arwani, Caroline A. Ross, Sahika Inal, Giovanni Costantini, Iain McCulloch

**Affiliations:** ^1^ Chemistry Research Laboratory University of Oxford Oxford UK; ^2^ Organic Bioelectronics Laboratory Biomedical Sciences Division King Abdullah University of Science and Technology (KAUST) Thuwal Thuwal Saudi Arabia; ^3^ School of Chemistry University of Birmingham Edgbaston Birmingham UK; ^4^ Department of Materials Science and Engineering Stanford University Stanford California USA; ^5^ Department of Chemical Engineering Massachusetts Institute of Technology Cambridge Massachusetts USA; ^6^ Andlinger Center For Energy and the Environment and Department of Electrical and Computer Engineering Princeton University Princeton NJ USA; ^7^ Department of Materials Science and Engineering Massachusetts Institute of Technology Cambridge Massachusetts USA; ^8^ School of Physics and Astronomy University of Birmingham Edgbaston Birmingham UK

**Keywords:** block copolymer, organic electrochemical transistor, rod‐coil block copolymer, semiconductor

## Abstract

Conjugated rod‐coil block copolymers provide a promising route to decouple the optimisation of ionic and electronic transport in organic electrochemical transistors (OECTs). Poly(3‐alkoxythiophene) (P3AOT)‐based rod‐coil block copolymers with precisely tuned polyethylene glycol (PEG), and poly(L‐lactide) (PLLA) coil blocks are synthesised through a modular approach combining Kumada coupling transfer polymerisation (KCTP) and Knoevenagel condensation. An evaluation of the thin film morphology and subsequent OECT performance of a range of rod‐coil copolymers reveals that device performance can be enhanced compared to the corresponding homopolymer, establishing rod‐coil block copolymers as a versatile material platform and providing fundamental insight for the use of block copolymer in OECTs.

## Graphical abstract

We present the first synthesis of poly(3‐alkoxythiophene) rod‐coil block copolymers as a new architecture for OMIECs. Incorporating PEG and PLLA coils can enhance device performance beyond the homopolymer. The figure‐of‐merit depends uniquely on coil length. Shorter chains compromise hydrophilicity, while longer chains can disrupt conductivity. Intermediate coils achieve an optimal balance, defining a design guideline within this architecture.

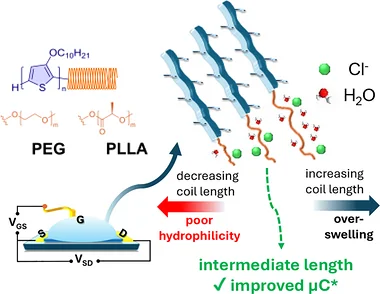

## 1 Introduction

Over the past decade, there has been a notable rise in the number of high‐performance semiconducting polymers employed in organic electrochemical transistors (OECTs) [1, 2, 3, 4, 5, 6, 7]. Such polymers have been classified as organic mixed ionic‐electronic conductors (OMIECs), exhibiting both ionic and electronic conductivity. Their soft, flexible, and hydrophilic nature allows them to be easily integrated into biocompatible scaffolds, opening exciting opportunities in electrochemical biosensing, wearable electronics, neuromorphic computing, and a broad range of related technologies [1, 8].

A conventional OECT device comprises a source, drain, and gate electrode. The source and drain electrodes are separated by a semiconducting polymeric active layer immersed in an aqueous electrolyte solution. Biasing the drain electrode with respect to the source creates a current flow from drain to source through the channel material (*I*_DS_). The applied gate voltage (*V*_GS_) injects ions to compensate the polaronic charge carriers. This ionic transport through the channel material regulates the charge carrier concentration and thereby its electronic conductivity. A key parameter benchmarking OECT performance is transconductance (*g*_m_), the derivative of the *I*_SD_ with respect to *V*_GS_, which is dependent on the product of two material‐related properties, namely the carrier mobility, *µ*, and the volumetric capacitance, C*, as shown in Equation (1).

(1) $$\begin{array}{c} \;g_{m}=\frac{Wd}{L}\;\mu C^{*}\left|V_{T}-V_{GS}\right| \end{array}$$

where *W*, *L* are width and length of the device channel, respectively; *d* is the film thickness and *V*_T_ the threshold voltage of the material.

Current state‐of‐the‐art polymeric materials utilised as OMIEC, such as the *p*‐type polythiophene‐based p(g2T‐TT) [9, 10] and *n*‐type polylactam‐based P‐75 [5], feature a coplanar conjugated polyarene backbone and hydrophilic oligoethylene glycol side chains, simultaneously favouring electronic charge transport through the conjugated backbone and ionic permeation through the glycol side chains. The use of side‐chain‐free conjugated ladder polymers such as poly‐benzimidazobenzophenanthroline (BBL) [11] and peNL [12] is an alternative strategy for designing high‐performance OMIEC. However, these polymers exhibit limited solubility in common organic solvents, which complicates both their synthesis and processing during device fabrication [13, 14]. A detrimental consequence of employing glycolated side chains, is that their hydrophilic nature allows the incorporation of water from the electrolyte, resulting in swelling and microstructure disruption during operation. Common strategies reducing the requirement for glycolation in OMIEC polymers includes incorporating a fraction of alkylated co‐monomer during polymerisation, or introducing side chains containing an alkyl spacer [2, 15, 16, 17].

Block copolymers are a versatile class of materials used in nanoscale morphology control due to their distinct self‐assembly behaviour. Conjugated rod‐coil block copolymers have garnered attention for their unique properties and application‐specific functionalities [18, 19]. In the OMIEC context, the conjugated backbone block is labelled as “rod” due to its rigidity, while other flexible polymer subunits are referred to as “coil”. In an idealised picture, block copolymer morphology is governed by the Flory‐Huggins parameter (χ) and the volume ratio of the component blocks (*f*) [20, 21]. In conjugated rod‐coil block copolymers, the stark dissimilarity in molecular composition between the conjugated rod segment and the flexible coil block results in a strongly positive χ parameter. Since χ specifies the degree of incompatibility between the block components, a positive and large value of χ favours microphase separation of the sub‐blocks. Formation of ordered nanostructures is highly dependent on the volume fraction *f* [22]. To minimise interfacial contact, curvature between the segregated nanodomain decreases as the volume fraction approaches symmetry (*f* » 0.5). Consequently, diblock copolymer systems exhibit a sequence of equilibrium morphologies with increasing block symmetry, transitioning from body‐centred cubic spheres to hexagonally packed cylinders, to bicontinuous gyroid structures, and ultimately to lamellae networks [22, 23]. This enables many applications such as patterning [24], drug encapsulation [25, 26], optoelectronics [27, 28, 29], and photocatalysis [30].

In conjugated rod‐coil block copolymers, the rigid conjugated rod backbone may result in aggregation via strong π‐π interaction, leading to deviations from ideal morphology. This can be further influenced by increase in the polydispersity (PD) of the polymer block, leading to increase in size of microphase‐segregated domains, altering phase transitions and ordering‐disordering temperatures [31]. Hence, the overall morphology of block copolymer film is governed by the combination of microphase segregation, crystallisation behaviour of each block as well as their molecular weight distribution. To date, using conjugated rod‐coil block copolymer in OECT device remains relatively unexplored, and a structure‐activity relationship between type of coil block, self‐assembly, and electrical performance in mixed transport applications is not yet clearly understood. However, the potential to decouple the electronic transport, confined to the conjugated block domains, from ionic transport, localised within the coil blocks, provides a platform for studying mixed transport phenomena in a fundamentally new manner.

Herein, we report a series of poly(3‐dodecyloxylthiophene) (P3DOT)‐based block copolymers as active mixed transport semiconductors for OECT devices. Polyether and polyester coil blocks, namely polyethylene glycol (PEG) and poly(L‐lactide) (PLLA), were incorporated to tune hydrophilicity and ionic uptake. The coil blocks were shown to strongly influence ion injection and swelling behaviour. Complementary OECT measurements revealed the influence of these properties on both volumetric capacitance and transconductance values, illuminating how differences in block copolymer composition can govern the balance between ionic and electronic transport.

## 2 Result and Discussion

### 2.1 Preparation of Block Copolymers

P3DOT, shown in Figure 1a, was chosen as the conjugated “rod” block. Pristine P3DOT homopolymer can be synthesised with narrow polydispersity and is readily soluble, with a DP_n_ of 53, as determined by ^1^H NMR spectroscopy (Figure S14), in good agreement with the corresponding *M*_n_ of 15.0 kDa measured by gel permeation chromatography (GPC). The electron‐rich conjugated backbone facilitates effective charge injection and better stabilises the injected hole polarons. Previous literature has featured several strategies to prepare rod‐coil block copolymers. These include further polymerisation from a conjugated polythiophene macroinitiator [32], or modularly coupling the functionalised conjugated precursor with a coil fragment through copper‐catalysed cycloaddition (CuAAC) reactions [33, 34]. However, these conventional methods proved ineffective for coupling P3DOT. Moreover, alkyne‐functionalisation of P3DOT resulted in substantial formation of insoluble product, possibly owing to homocoupling side‐reaction. Catalyst screening (Table S1) revealed that Ni(IPr)(PPh_3_)Cl_2_ produced P3DOT macroinitiators with an optimal combination of high‐molecular weight and narrow polydispersity. These polymer backbones terminated predominantly with H/Br end groups (Figure S8). The effectiveness of the catalyst is attributed to the steric and electronic properties of the NHC ligand, which reinforces the stability of the catalyst‐polymer π‐complex with a stronger three‐centre, four‐electron interaction, while shielding the propagating chain from undesired chain transfer reactions through catalyst dissociation [35].

**FIGURE 1:**
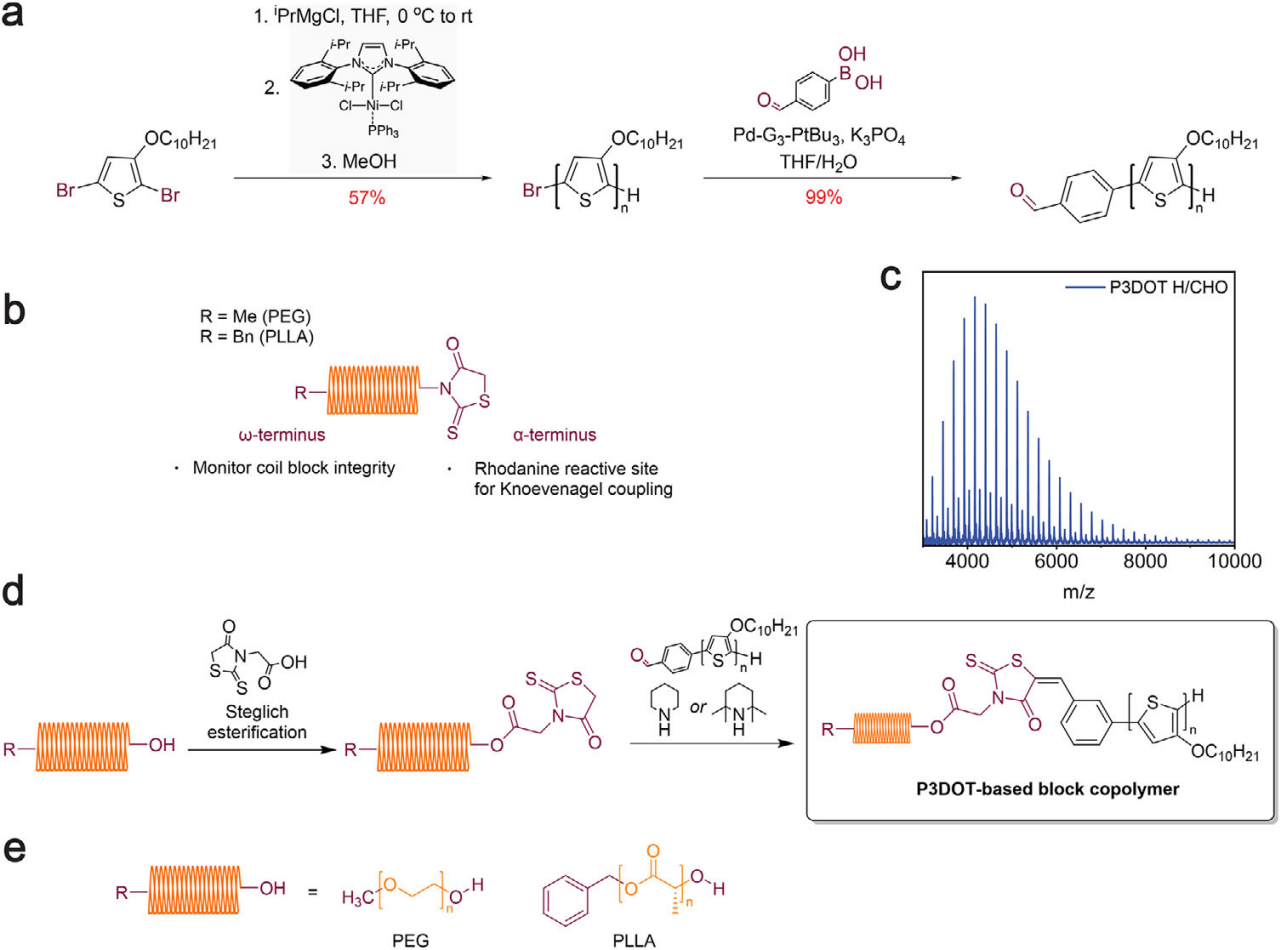
(a) Synthesis of aldehyde‐terminated P3DOT. (b) End‐group design of the coil block. (c) Matrix‐assisted laser desorption/ionization time‐of‐flight mass spectrometry (MALDI‐TOF‐MS) spectrum of P3DOT H/CHO. (d) Synthesis of functionalized coil block and P3DOT‐based block copolymer. (e) Structures of coil block.

The H/Br terminated P3DOT precursor was then further functionalised through a Suzuki‐Miyaura reaction to introduce an aldehyde end‐group (Figure 1a,c), which proceeded with nearly quantitative conversion. Pd content from the catalyst was controlled by metal scavenging, which was further characterised by inductively coupled plasma mass spectrometry (ICP‐MS) to be 3.8 ppm (Section S9). The average mole fraction of the aldehyde in the polymer chain was then quantified by ^1^H NMR (Figure S14).

Two polar coil blocks, PEG and poly(L‐lactide) (PLLA), were selected to enhance hydrophilicity and thereby improve ionic conductivity of the block copolymers. Owing to their distinct intermolecular interactions and conformational flexibility, these coil segments were expected to be highly mismatched with the conjugated rod, potentially favouring self‐assembly into segregated microstructures. The coil length primarily influenced swelling behaviour, ionic uptake, and molecular ordering. To probe these effects systematically, coil blocks with varying number‐average degrees of polymerisation (DP_n_) were prepared (Table 1).

**TABLE 1:** Molecular weight properties of all P3DOT rod‐coil block copolymers. The number‐average degree of polymerization (DP_n_), molecular weight (M_n_), and polydispersity (PD) were obtained from GPC analysis.

Block copolymer	Coil DP_n_	Coil PD	Block copolymer M_n_ (kDa)	Block copolymer PD
P3DOT	0	N/A	15.0	1.3
P3DOT‐b‐PEG‐10	10	1.1	16.2	1.2
P3DOT‐b‐PEG‐18	18	1.2	16.5	1.5
P3DOT‐b‐PEG‐40	40	1.2	17.4	1.5
P3DOT‐b‐PEG‐80	80	1.3	19.5	1.4
P3DOT‐b‐PLLA‐34	34	1.4	16.4	1.4
P3DOT‐b‐PLLA‐65	65	1.5	18.3	1.4

Inspired by prior studies featuring highly efficient Knoevenagel condensation for synthesis of nonfullerene acceptors [36, 37, 38], we developed a Knoevenagel‐based coupling of the rod and coil blocks, owing to its excellent functional‐group tolerance, high reaction efficiency, wide solvent compatibility and metal‐free nature. To enable reactivity with the aldehyde moiety on the polythiophene, PEG and PLLA were functionalised with a rhodanine moiety (Figure 1b,d,e) on α‐alcohol terminus. In addition, the ω‐terminus was decorated either with a methyl group (for PEG) or benzyl group (for PLLA). ^1^H NMR signal of ω‐terminus not only confirmed the coupling efficacy but also verified the structural integrity of the coil block following Knoevenagel coupling, since any fragmentation of the coil block, regardless the extent, would remove the ω‐terminus.

Reaction optimisation was performed with P3HT dialdehyde. Base screening (Table S2 and Figure S9) demonstrated that piperidine was highly effective for the coupling with PEG, possibly owing to the activation of the rod‐block via an iminium intermediate [39]. However, this condition also promoted base‐mediated degradation of the ester moiety in PLLA, as evidenced from the loss of the ω‐terminus. To mitigate this sensitivity, a sterically hindered base catalyst, 2,2,6,6‐tetramethylpiperidine was selected, which successfully preserved the PLLA block during coupling. With the optimised condition, PEG fragments with DP_n_ of 10, 18, 40, and 80, as well as PLLA fragments with DP_n_ of 33 and 65 were anchored to the P3DOT macroinitiator. The success of rod‐coil coupling was marked by significantly reduced retention time in GPC (Figure 2a,b).

**FIGURE 2:**
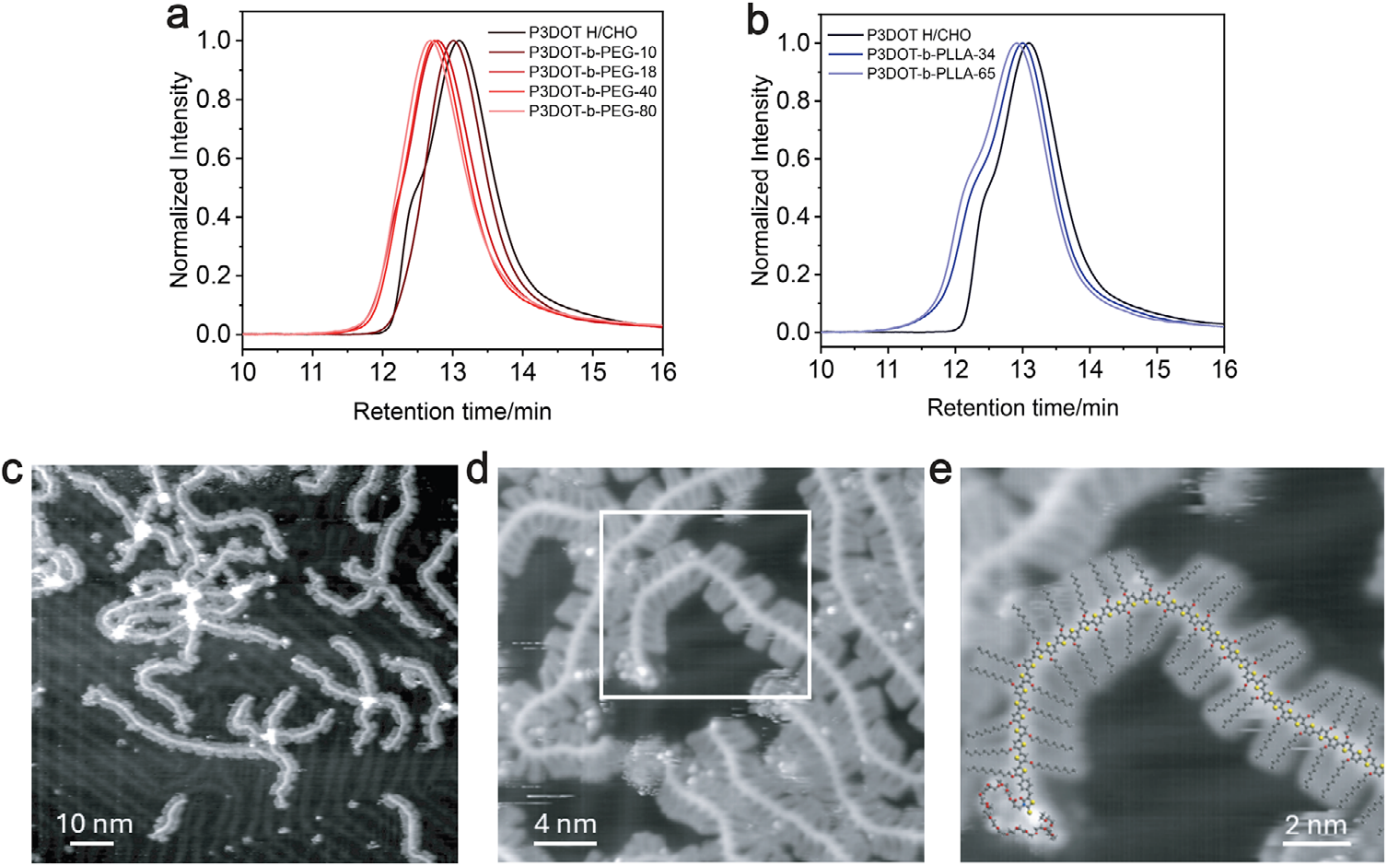
GPC trace of (a) P3DOT‐b‐PEG series and (b) P3DOT‐b‐PLLA series. (c) Large‐scale STM image of P3DOT‐53‐b‐PEG‐18 deposited by ESD on Au(111). (d) Higher‐resolution image showing details of lateral polymer interactions. (e) Zoom into the region marked by the white rectangle in b, with the molecular structure of a single polymer chain superposed.

### 2.2 Block Copolymer Imaging With Scanning Tunneling Microscopy

The structure of P3DOT‐b‐PEG‐18 was imaged by electrospray deposition scanning tunnelling microscopy (ESD‐STM) [40, 41]. Large‐area STM images (Figure 2c) show that, while many polymer chains exhibit lengths consistent with the average *M*_n_ value determined by NMR, chains of both shorter and longer length are also observed, reflecting the dispersity measured by GPC (Table 1). High‐resolution STM images (Figure 2d,e) clearly resolve the polythiophene backbone and the OC_10_H_21_ straight side chains of the P3DOT rod component lying flat on the substrate (dark background). One of the chain termini displays a short and less well‐defined structure, which is assigned to the PEG‐18 coil coupled to the P3DOT ω‐terminus. The PEG chains consistently appear highly coiled, which is attributed to their high conformational flexibility [42], and they occasionally move under the STM tip during scanning.

The entire P3DOT‐b‐PEG‐18 polymer chains exhibit a high degree of conformational flexibility on the surface, with isolated molecules typically adopting curved conformations (Figure 2c) [43], as evaluated by fitting molecular models of the polymer chains to high‐resolution STM images at the single‐monomer level [40, 44, 45]. No long‐range ordering is observed and only in regions of locally increased molecular density do neighbouring P3DOT backbones approach each other, reaching minimum interchain distances of approximately 2.6–2.7 nm. This spacing is consistent with the absence of side chain interdigitation and with an average angle of ∼30°–35° between the backbone and the alkoxy side chains, in close agreement with structural motifs previously reported for polythiophene thin films [46, 47]. In most cases, however, larger interchain separations of ∼3–4 nm are observed, which can be attributed both to the nonlinear backbone conformations and to side chains adopting more perpendicular orientations relative to the backbone (Figure S34).

### 2.3 Microstructure of Block Copolymers

The microstructure of the block copolymers was further analysed by grazing incidence wide‐angle x‐ray scattering (GIWAXS) on a silicon substrate. All polymer films exhibited pronounced anisotropy with an “edge‐on” backbone orientation (Figures 3a–c and S46), with three orders of scattering from lamellae (h00), stacked in the out‐of‐plane (OOP) direction with (100) *d*‐spacings from 23 to 25 Å (Table S3), which is compatible with the values obtained from STM imaging for on‐surface assembly (Figure 2c–e). The in‐plane (010) peak was ascribed to π‐π stacking. Upon introduction of the coil block, the (010) *d*‐spacing expands from 3.5 to about 3.9 Å. In the in‐plane directions, the (010) scattering was fitted with the superposition of 2 Gaussian functions, which correspond to π‐π scattering from amorphous and semicrystalline morphologies. The molecular ordering of the block copolymers was estimated based on the total integrated intensity of the (010) scattering from sample films with nominally identical thickness. The P3DOT‐b‐PEG‐18 and P3DOT‐b‐PLLA‐34 polymers exhibit the highest degree of ordering in the (010)‐direction and correspondingly show the best charge carrier mobility (Figure 5d,e). The influence of thermal treatment on the microstructure was examined by annealing the block copolymer samples at 150°C. The observed contraction in the (100) *d*‐spacing is likely due to side chain reorganisation, facilitated by their increased mobility at elevated temperatures. However, similar to P3HT [46], side chain interdigitation is not observed. Overall, thermal annealing reveals significant change on surface morphology of the block copolymer, which is further supported by atomic force microscopy (AFM) images of P3DOT‐b‐PEG‐18 thin films acquired before and after annealing (Figure S35). As shown by differential scanning calorimetry (DSC, Figure S50), all block copolymers possessed good thermal reversibility with no significant change in melting endotherm and crystallisation exotherm following heating to 260°C. Interestingly, the coil component of most block copolymer remains vitrified, implying that the overall melting and crystallisation of the block copolymer is dominated by phase transitions in the rod block. The only exception was P3DOT‐b‐PEG‐80. This could be attributed to the long and flexible PEG‐80 chains, which promotes self‐assembly of the coil domains and leads to the emergence of coil crystallisation upon annealing.

**FIGURE 3:**
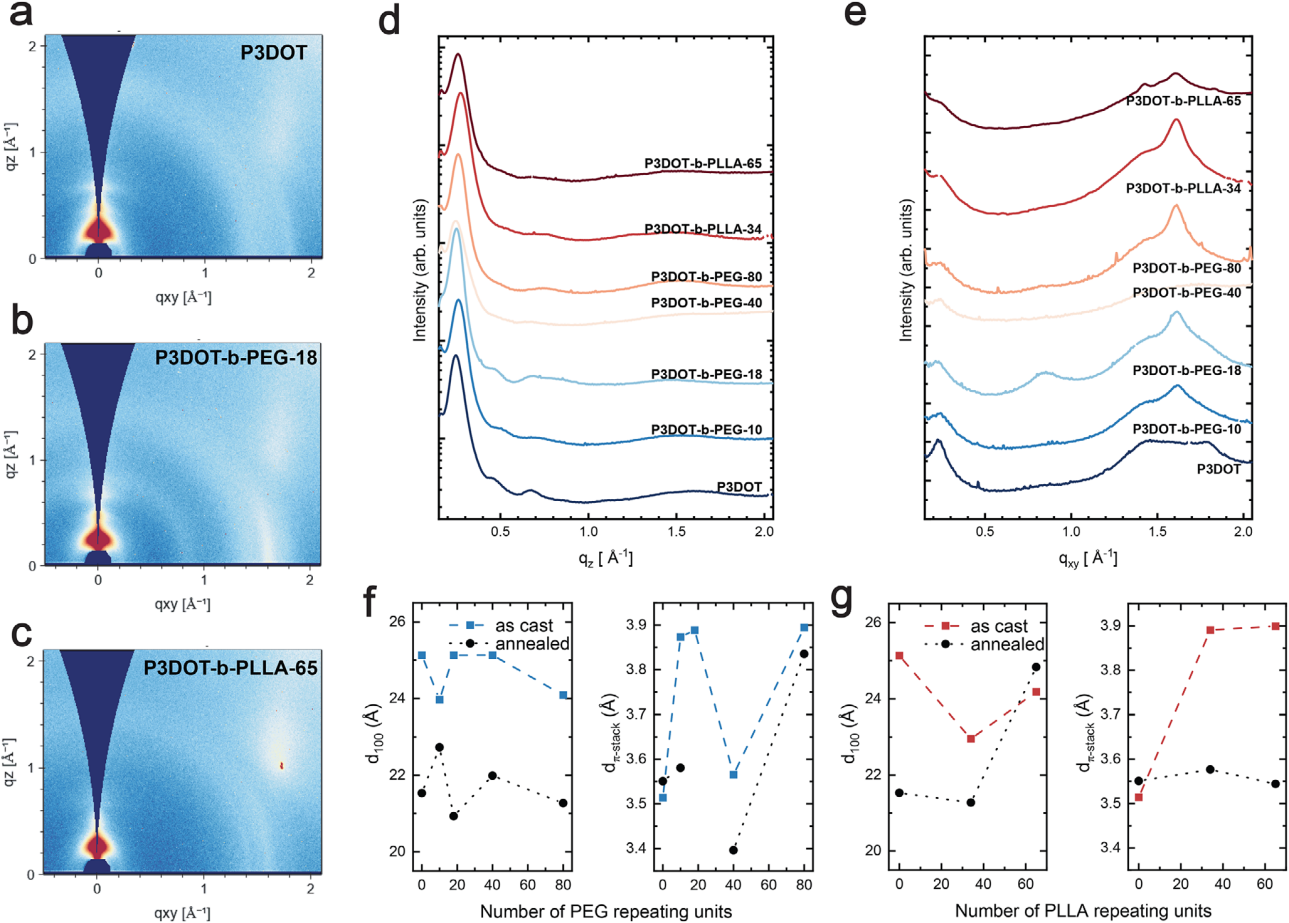
2D GIWAXS of as‐casted (a) P3DOT, (b) P3DOT‐b‐PEG‐18, (c) P3DOT‐b‐PLLA‐65. (d) Out‐of‐plane and (e) in‐plane 1D linecuts of P3DOT‐b‐PEG and P3DOT‐b‐PLLA series. Lamellar (100) and p‐stacking distances for the (f) P3DOT‐b‐PEG series and (g) P3DOT‐b‐PLLA series.

### 2.4 Optical and Electrochemical Properties

The optical and electronic properties of the block copolymers were investigated using photoemission spectroscopy in air (PESA) (Figure S29), ultraviolet‐visible (UV‐Vis) spectroscopy (Figure S30), and cyclic voltammetry with TBAPF_6_ electrolyte in acetonitrile (Figure S31). These measurements showed minimal variations in ionisation potential and optical bandgap across all polymers. This suggests that the electronic properties are not strongly affected by the coil length or composition. In fact, all block copolymers exhibited an absorption maximum around 650 nm, with an additional shoulder peak observed around 700 nm, previously attributed to the vibronic fine structures [48]. The impact of electrochemical doping was investigated with spectro‐electrochemical measurement in 0.1 M NaCl aqueous electrolyte solution (Figure S33). Spectroelectrochemical response of the P3DOT‐*b*‐PEG‐18 is shown in Figure 4a with an external bias of ‐0.2 to 0.7 V under ambient condition. Under applied bias, the intensity of π‐π* absorption around 650 nm gradually decreased, with the concomitant emergence of a new absorption peak near 960 nm, attributed to formation of polaronic carriers. An applied reverse bias of ‐0.2 V restored the π‐π* peak at 650 nm and diminished polaronic absorption, confirming the electrochemical stability of all block copolymers. Surprisingly, extending the PEG coil length did not significantly lower the onset voltage required for electrochemical doping (Figure 4b). This indicates that the threshold voltage for ion injection is largely dictated by the conjugated rod backbone, with coil incorporation exerting only a minor influence.

**FIGURE 4:**
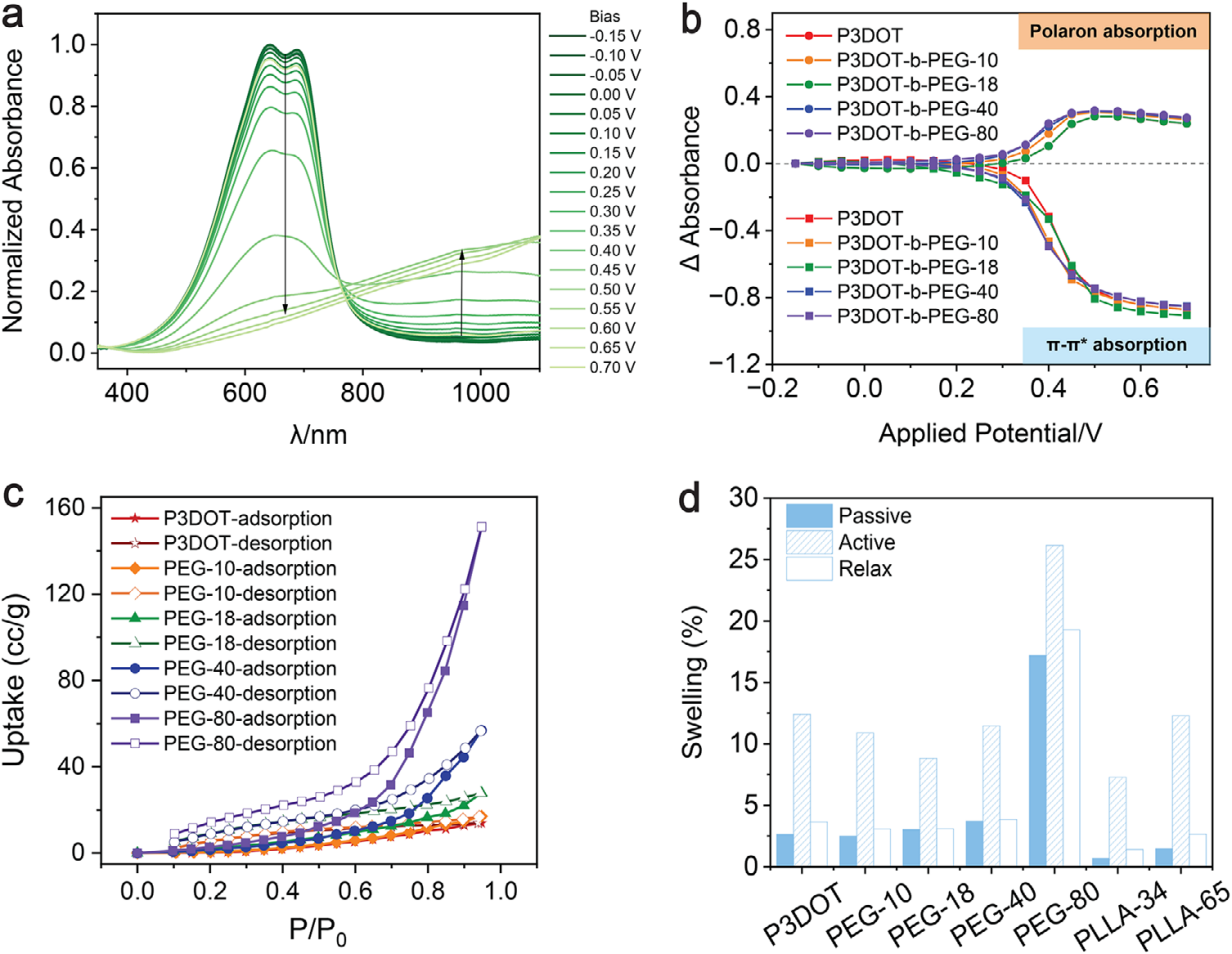
(a) Spectroelectrochemistry (SEC) of P3DOT‐b‐PEG‐18. (b) Absolute changes in normalized polaron and π–π* absorption under different applied bias. (c) Moisture Brunauer–Emmett–Teller plot of the P3DOT‐b‐PEG series. (d) Swelling of P3DOT‐b‐PEG and P3DOT‐b‐PLLA from EQCM‐D measurements.

### 2.5 Hydrophilicity and Swelling Properties

The hydrophilicity of the P3DOT‐*b*‐PEG block copolymers was investigated using water vapour BET analysis (Figure 4c). The absorption process in all samples reveals a Type III isotherm [49], arising from clustering of water vapour around more hydrophilic surfaces on the bulk powder [50]. Under high humidity, longer PEG coil resulted in greater uptake of moisture, consistent with enhanced hydrophilicity. Interestingly, P3DOT‐*b*‐PEG‐40 and P3DOT‐*b*‐PEG‐80 showed incomplete desorption at the end of the cycle. This is likely caused by irreversible swelling, creating voids with trapped moisture.

To understand the swelling behaviour of the polymer films, EQCM‐D (Figure S41) was used to quantify the change in film thickness following ion uptake (Figure 4d). In all cases, the block copolymers present low dissipation factor, indicating rigid thin‐film properties. The passive swelling of P3DOT‐*b*‐PEG increased with longer PEG chain, consistent with the greater hydrophilicity of the longer coil blocks. The active swelling of the polymer films showed a more nuanced dependence on DP_n_ of the coil block. Despite the amorphous and hydrophilic nature of the PEG coil, incorporation of a short PEG chain reduced the active swelling from 12.4% in the P3DOT homopolymer to 8.8% in P3DOT‐*b*‐PEG‐18. Further extension of PEG coil reversed this trend, leading to increasing passive and active swelling in P3DOT‐*b*‐PEG‐40 and P3DOT‐*b*‐PEG‐80, reflecting higher water uptake favoured by greater hydrophilicity and higher proportion of flexible coil domain on‐film. These results indicate that longer PEG chains led to excessive film expansion, while that of intermediate length better preserved the film structure during active swelling. P3DOT‐*b*‐PLLA block copolymers also followed this pattern, with passive swelling values of 0.7%–1.5% and active swelling of 7.3%–12.3%, which were both lower than the PEG counterparts with similar chain length and even the P3DOT homopolymer itself. The reduced swellability of P3DOT‐b‐PLLA is attributed to the lower hydrophilicity of PLLA relative to PEG, evidenced from its greater contact angle [51], as well as improved swelling ratio of a PLLA‐PEG blend [52] compared to PLLA homopolymer [53]. Its more ordered, semicrystalline structure [54] could hence better impede water permeation.

### 2.6 Organic Electrochemical Transistors

OECTs with channels fabricated from each block copolymer were characterised in 0.1 M NaCl electrolyte using an Ag/AgCl pellet as the gate electrode. All devices operated in enhancement mode. The geometry‐normalised OECT characteristics and figures of merit are presented in Figure 5a–c and Table 2, respectively. Interestingly, without the addition of a coil block, the pristine P3DOT backbone demonstrated a µC* of 113 Fcm^−1^Vs^−1^, comparable to many glycolated OMIECs [55]. The hydrophobic nature of the P3DOT side chains led to slow ionic transport, evidenced by the hysteretic nature of the transfer curve and long turn‐on response times. Incorporation of the coil block introduced a more complex dependence of device performance on coil length. To separate the relative contribution of ionic and charge carrier transport, C* was extracted from the slope of the injected charge density as a function of applied potential (Figure S42). As shown in Figure S43, increasing the PEG coil length up to a DP_n_ of 40 led to higher C*, which is consistent with its better ion affinity. However, C* plateaued at P3DOT‐b‐PEG‐80 despite increased swelling. Interestingly, the P3DOT‐b‐PLLA series showed higher C* than P3DOT‐*b*‐PEG, which we attribute to more effective ion percolation facilitated by the ester functionalities in PLLA, whose increased polarity promotes ion uptake within the polyester domains, consistent with prior molecular dynamics simulation [56]. In addition, dynamic response measurements (Figures S44 and S45) show that P3DOT‐*b*‐PEG exhibits faster response times with increasing chain length, whereas both P3DOT‐*b*‐PLLA polymers display intermediate response times between those of the P3DOT homopolymer and P3DOT‐b‐PEG‐18, indicating that C* and ion uptake kinetics are not directly correlated.

**FIGURE 5:**
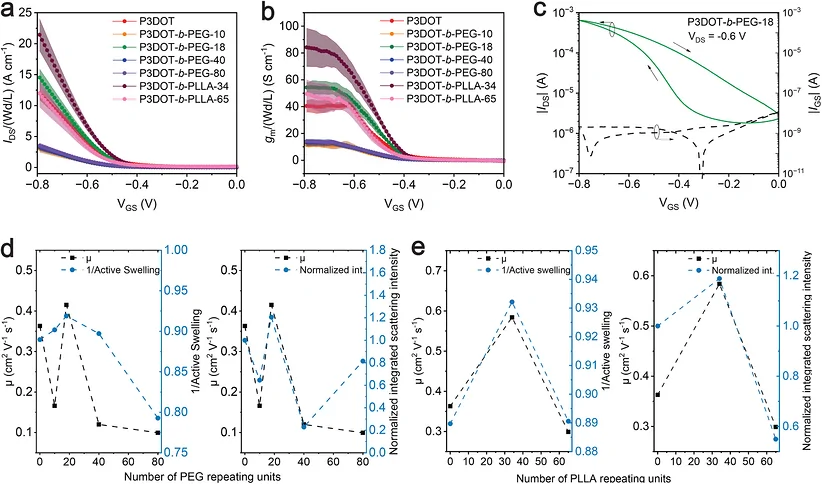
Geometry normalised (a) transfer (*I*_DS_) and (b) transconductance (*g*_m_) curves of the block copolymers obtained from OECT characterisation, the error bands represent at least five independent OECT channels. (c) Transfer curve |*I_DS_* |(blue line, left axis) and gate leakage current |*I_GS_* |(dark dash, right axis) of P3DOT‐*b*‐PEG‐18‐based OECT. *V*_DS_ = ‐0.6 V with a scan rate of 10 mV/s for the OECT measurements. The arrows indicate the direction for forward and backward voltage sweep. Comparison of µ*, 1/active swelling and normalised integrated scattering intensity in IP direction of (d) P3DOT‐b‐PEG series, (e) P3DOT‐b‐PLLA series.

**TABLE 2:** OECT figure of merit of all P3DOT‐b‐PEG block copolymers.

Block copolymer	V_TH_ (V)^a^	µC* (Fcm^−1^V^−1^s^−1^)^b^	C* (Fcm^−^^3^)^c^	µ (cm^2^V^−1^s^−1^)^d^	ON/OFF response time (s)^e^	ON‐OFF ratio^f^
P3DOT	−0.40	110 ± 11	310 ± 40	0.36 ± 0.06	3.5/0.29	1.9 × 10^2^
P3DOT‐b‐PEG‐10	−0.39	63 ± 1.3	370 ± 13	0.17 ± 0.01	2.7/0.18	3.2 × 10^3^
P3DOT‐b‐PEG‐18	−0.36	150 ± 22	370 ± 30	0.42 ± 0.07	1.7/0.38	5.3 × 10^3^
P3DOT‐b‐PEG‐40	−0.37	52 ± 2	430 ± 80	0.12 ± 0.02	1.3/0.17	5.1 × 10^3^
P3DOT‐b‐PEG‐80	−0.37	40 ± 3	400 ± 70	0.10 ± 0.02	0.70/0.042	5.4 × 10^3^
P3DOT‐b‐PLLA‐34	−0.36	240 ± 30	420 ± 40	0.58 ± 0.09	2.9/0.24	3.7 × 10^2^
P3DOT‐b‐PLLA‐65	−0.38	150 ± 25	510 ± 50	0.30 ± 0.05	1.7/0.14	7.5 × 10^3^

^a^ Obtained from the slope of the (*I_DS_*)*0.5* versus V_GS_ curve.

^b^ Calculated based on the transconductance value extracted from transfer characteristics based on $g_{m}=\mu C^{*}\frac{Wd}{L}\;\left(V_{GS}-V_{th}\right)$ ^.^ The average values were calculated from at least 4 devices.

^c^ Determined by integrating the chronoamperometric current acquired during EQCM‐D measurements to obtain the injected charge, evaluated at the indicated potentials.

^d^ Calculated by dividing µC* by C*.

^e^ The response time was extracted from the bi‐exponential fitting of the OECT ON/OFF switch response curves.

^f^ Obtained by calculating *I_DS_* (max)/*I_DS_* (min).

The charge carrier mobility of each block copolymer was determined using Equation (1). To better understand its complex behaviour, *µ* was plotted alongside the active swelling ratio and the thickness‑normalised integrated GIWAXS scattering intensity in the in‑plane direction, which reflects local π–π stacking order. Overall, *µ* exhibits an approximate inverse correlation with the active swelling ratio (Figure 5d,e), consistent with earlier reports [57]. Among all samples, P3DOT‑b‑PLLA‑34 displayed the highest mobility of 0.58 cm^2^ V^−^^1^ s^−^^1^, coinciding with the lowest active swelling ratio of 7.3% (Figure 5e). A positive correlation was also observed between *µ* and enhanced π–π stacking, indicating that improved molecular ordering promotes charge transport. Notably, deviations from the main trends were observed in particular for P3DOT‐*b*‐PEG‐10 and P3DOT‐*b*‐PEG‐40, which exhibit lower *µ* than the P3DOT homopolymer despite reduced active swelling ratio; in these cases, *µ* correlates more closely with π–π stacking order.

The operational stability of the block copolymers is measured with pulsed operation stability cycling (Figure S51). As the PEG coil is extended from DP_n_ = 18 to 80, on‐current retention of the block copolymers decreases from 86% to 56%, mirroring the onset of excessive swelling, while the apparently complete retention of the P3DOT homopolymer (101%) reflects its incomplete switching within each pulse rather than superior stability. On the other hand, P3DOT‐b‐PLLA‐65 demonstrates better operational stability relative to P3DOT‐b‐PLLA‐34, which is attributed to the greater fraction of rigid, semicrystalline PLLA domains that suppress excessive swelling and reduces structural disruption during electrochemical cycling.

Overall, however, the data indicate that incorporating coil blocks of moderate chain length maximises *µ*, as demonstrated for P3DOT‐*b*‐PEG (DP_n_ of 18) and P3DOT‐*b*‐PLLA (DP_n_ of 34), where swelling behaviour and π–π stacking are simultaneously optimised. Owing to their moderately high C*, these materials also achieve an optimal µC*, reflecting a favourable balance between electronic and ionic transport. In terms of stability, P3DOT‑b‑PEG‑18 represents the most balanced design combining efficient transport with robust current retention, whereas the shorter PLLA analogue shows only moderate retention, suggesting that the stabilizing influence of semicrystalline PLLA domains becomes more pronounced at higher chain lengths.

## 3 Conclusion

This work demonstrates the use of a novel block copolymer molecular architecture for OMIEC design, based on a P3DOT rod backbone coupled to hydrophilic ether or ester coil blocks. While the pristine hydrophobic P3DOT polymer exhibits a respectable µC* (Table 2), its hysteretic transfer characteristics and slow response highlight the need for coil incorporation. Systematic variation of PEG and PLLA coil lengths shows that device performance is governed by the interplay between swelling behaviour, ionic uptake and π‐π stacking order. Notably, P3DOT‐*b*‐PLLA‐34 achieves the optimal µC* (240 Fcm^−1^Vs^−1^), together with the lowest active swelling (7.3%), and the highest π‐π scattering intensity. Overall, incorporating coil blocks of intermediate chain length provides the most favourable balance, maximising charge carrier mobility while maintaining sufficient ionic transport. By elucidating how coil chemistry and length modulate both ionic and electronic contributions, this study establishes structural guidelines of block copolymer OMIECs, and advances fundamental understanding required for next‐generation OECT materials.

## 4 Experimental Section

### 4.1 Materials

All reagents were purchased from commercial suppliers. P3HT dialdehyde was prepared according to published procedures [58]. Synthesis of 2,5‐dibromo‐3‐decyloxythiophene monomer was adapted from a previous report [10]. ^1^H and ^13^C nuclear magnetic resonance (NMR) spectra were taken on a Bruker AVIII HD 400 or AVIII HD 500 spectrometer. High resolution ESI mass spectra were recorded on a Bio‐Accord LC‐MS system. MALDI spectra were taken on Autoflex MALDI‐TOF/TOF instrument with linear mode. Analytical GPC was taken on either (chloroform, via RI‐detector and UV‐detector with polystyrene standard) was performed on either Agilent 1200 Series GPC‐SEC system using HPLC chloroform eluent at 40°C or Agilent 1260 Infinity II using HPLC chlorobenzene eluent at 80°C.

### 4.2 Synthesis of Monomer, Polymer and Block Copolymer

The synthetic step of monomer, polymer and block copolymers were detailed in the supporting information. (Section S1.4, Supporting Information). The ^1^H NMR spectroscopy, ^13^C NMR spectroscopy and mass spectrometry results of the small‐molecule products in each step were shown in Figures S11 and S12. MALDI‐TOF‐MS, 1H NMR spectroscopy and GPC figure of the polymer and block copolymers were shown in Figures S13–S27.

### 4.3 Electrospray Deposition—Scanning Tunnelling Microscopy

Monolayer samples of P3DOT‐b‐PEG‐18 were prepared by electrospray deposition (ESD) using a four‐stage Molecularspray Ltd. system. The polymer was dissolved in chlorobenzene (∼0.04 g/L) and diluted with methanol in a 4:1 volume ratio. The resulting solution was electrosprayed onto an atomically flat single crystal Au(111) substrate held at room temperature under high vacuum conditions. Prior to deposition, the substrate was cleaned by repeated cycles of argon ion sputtering followed by annealing at 500°C in ultra‐high vacuum (UHV). STM measurements were performed at ∼10‐10 mbar and 77 K using a low‐temperature STM (CreaTec Fischer & Co. GmbH).

For the analysis of the STM images, molecular models of individual monomers were geometry optimized using the MMFF94 force field in the Avogadro software and manually superimposed onto high‐resolution STM images using the LMAPper software to match the correct scale of the images.

### 4.4 Atomic Force Microscopy

AFM images were acquired using an Asylum Cypher‐L AFM system (Oxford Instruments–Asylum Research, Santa Barbara, USA). Measurements were performed under ambient conditions in tapping mode, employing a Multi75‐Al‐G cantilever (Budget Sensors, Bulgaria) with a fundamental resonance frequency of approximately 75 kHz. To achieve high‐resolution imaging, the cantilever was driven at its second eigenmode, corresponding to a resonance frequency of approximately 500 kHz. The amplitude sensitivities of the first and second eigenmodes, equal to 64 nm/V and 118 nm/V, respectively, were determined using the thermal calibration method implemented in the Ergo acquisition software. Asylum Cypher‐S AFM (Oxford Instruments–Asylum Research, Santa Barbara, USA). was also applied to capture the phase images. NC160 (NanoandMore, USA) with a resonance frequency of 300 kHz and a spring constant of 26 N/m was used to analyse the samples with tapping mode at room temperature. Samples were prepared on p‐doped silicon substrates by spin‐coating at 1500 rpm for 1 min from a 5 mg/mL solution of P3DOT‐53‐b‐PEG‐18 in chloroform, followed by thermal annealing at 150°C for 30 min under a nitrogen atmosphere. All samples were analysed immediately after preparation.

### 4.5 Cyclic Voltammetry

Cyclic voltammetry measurements were carried out under ambient air and temperature conditions using AutoLab PGSTAT101 machine with Metrohm Autolab NOVA software version 2.1. A three‐electrode configuration was used for all measurements, with a polymer‐deposited ITO substrate as working electrode and a platinum mesh wire as counter electrode. For measurement conducted in 0.1 M aqueous NaCl supporting electrolyte, a BASi Ag/AgCl (3 M NaCl) Reference Electrode (MF‐2052) was used as reference electrode. For measurement using 0.1 M TBAPF_6_ solution in acetonitrile as electrolyte, a BASi Ag/Ag+ nonaqueous reference electrode (MW‐1085) filled with 0.01 M AgNO3 and 0.1 M TBAP solution was used and calibrated against ferrocene/ferrocenium redox couple (Fc/Fc+). All cyclic voltammograms were recorded with a scan rate of 100 mVs‐1. Ionization potentials were estimated based on equation below.

(2) $$\begin{array}{c} IP=4.8\;V+E_{ox}-E_{\frac{Fc}{Fc^{+}}}\; \end{array}$$

herein, E_ox_ refers to the oxidation onset potential of polymer film using 0.1 M TBAPF_6_ in acetonitrile as an electrolyte and E_Fc/Fc+_ to the half‐wave potential of the ferrocene/ferrocenium couple in identical conditions.

### 4.6 UV‐Vis Spectroscopy

UV‐Vis absorption spectra were collected under ambient air and temperature conditions using a UV‐1800 Shimadzu spectrophotometer with UV‐Probe 2.42 software. Thin‐film UV‐Vis absorption spectra were measured from polymer‐coated glass substrates over a wavelength range of 350–1100 nm. The optical bandgap was evaluated based on the onset wavelength of absorption.

### 4.7 Spectro‐Electrochemistry

Spectro‐electrochemistry was conducted in a “Cappuccino PEC Cell” [59] placed in a UV‐1800 Shimadzu spectrophotometer. A three‐electrode configuration, identical to that used in cyclic voltammetry, was connected in the cell. During each measurement, a uniform, continuous potential was applied using an AutoLab PGSTAT101 for 10 s to the polymer‐coated ITO working electrode to allow equilibration before the optical absorption was recorded.

### 4.8 Photoemission Spectroscopy in Air (PESA)

PESA spectra were recorded using a KP Technology APS02 system over wavelengths of ca. 180 – 280 nm on a polymer‐coated glass slide. All photoemission measurements were performed under an applied bias of ‐70 V. The ionization potential was evaluated from the onset energy level with respect to the cube root of the photoemission intensity.

### 4.9 Water Vapour Adsorption Isotherm

All gases used for sorption analysis were supplied by BOC at a purity of ≥99.999%. The polymers were precipitated into methanol following Soxhlet purification (or washed if insoluble), as described in the polymer synthesis section, and then dried in a vacuum oven at 50°C overnight. The hydrophilicity of the materials was evaluated from the volume of water vapour uptake per unit mass using adsorption isotherm with H_2_O as the adsorbate. Approximately 50 mg of the polymer sample was loaded into an Anton Paar Autosorb iQ‐XR gas sorption analyser. The materials were heated at 110°C for 18 h under vacuum on the apparatus prior to measurement to remove residual moisture. The water sorption isotherm was measured at 25°C in a water bath.

### 4.10 Grazing Incidence Wide‐Angle X‐Ray Scattering

Grazing incidence wide‐angle x‐ray scattering (GIWAXS) measurements were conducted at the Stanford Synchrotron Radiation Lightsource, SLAC National Accelerator Laboratory, Beamline 11–3. The energy of the x‐ray beam was 12.7 keV. All samples were measured at an incidence angle of 0.12°. Scattering intensity was collected using a Rayonix MX225 CCD 2D area detector. The sample‐to‐detector distance was calibrated using a LaB6 polycrystalline standard. All data are reported in terms of the scattering vector, q = (4π/λ)sin(θ), where θ is the scattering angle. Data were processed using a custom Python code4. Linecuts were integrated over in the range 5–30° and 65–87° for out‐of‐plane and in‐plane directions, respectively.

### 4.11 Differential Scanning Calorimetry

The melting and crystallization behaviours of the block copolymers were evaluated using differential scanning calorimetry (DSC) with TA Discovery 25‐Auto under a nitrogen atmosphere. The temperature for the DSC thermal history was varied from 0°C to 260°C at a rate of 10°C/min. Data analysis was performed using Trios software.

### 4.12 OECT Device Fabrication

The OECTs were fabricated on 4‐inch glass wafers (UniversityWafer) based on previously established protocols [60] using standard lithography and parylene C peel‐off techniques. To pattern the gold electrodes (source and drain), the photoresistor AZ5214 was spin‐coated on the pre‐cleaned glass wafers and exposed by using the UV light through an EVG 62100 contact aligner. The photoresistor was developed by an MF‐319 developer. Next, 10 nm chromium and 100 nm gold were deposited sequentially by magnetron sputtering using an ESCRD4 purchased from Equipment Support Company Ltd. A 1.7 µm insulator layer of parylene C was vaporized using an SCS Labcoater 2 and adhered to substrates employing (trimethylsilyl)propyl methacrylate as an adhesion promoter. Between the deposition of the second sacrificial layer of parylene C, an aqueous soap was spin‐coated to promote the peel‐off process. A thick photoresistor AZ10XT was spin‐coated and developed to define the channel and gate area using the mask aligner. Finally, the channel and gate area were exposed by using a reactive ion etcher, Oxford Instruments Plasmalab 100 – ICP 380. The polymer thin films were spin‐coated (1500 rpm for 60 s) from a chloroform solution with a concentration of 5 mg mL‐1 on the substrates. Then the sacrificial layer of parylene C was peeled‐off to define the active areas. The device was washed by DI water to remove the Micro‐90 solution on the insulator layer and dried by a gentle N_2_ flow.

### 4.13 OECT Device Characterization

All measurements were performed under ambient atmospheric conditions. The electrical characterization of devices was done using a Keithley 2636A source meter unit operated by customized LabVIEW software or Kickstart software.

### 4.14 Volumetric Capacitance Measurement

The injected charge density of polymer films was determined by integrating the current obtained from chronoamperometry measurements during e‐QCMD experiments according to established protocols [11, 61]. Square voltage pulses (60 s each) were applied stepwise from 0 to 0.8 V versus Ag/AgCl reference electrode. The corresponding current response was recorded and integrated over time to yield the injected charge evolution. To eliminate contributions from faradaic processes, the final 10 s of each pulse were linearly fitted, and the intercept value was taken as the total injected charge for that voltage step. The injected charge density was then plotted as a function of the applied potential, and the slope of the resulting curve was used to calculate the volumetric capacitance. Alternatively, the volumetric capacitance was obtained from integrating the gate current during transient measurement.

### 4.15 Operando Electrochemical Quartz Crystal Microbalance With Dissipation Monitoring (eQCM‐D)

Measurements were performed using the QSense Analyzer, a QSense Window Electrochemistry Module (QWEM 401) from Biolin Scientific, and gold with titanium adhesion layer sensors (QCM5140TiAu120‐050‐Q) from Quartz Pro. The analyzer measured the shift and dissipation of the first, third, fifth, seventh, nineth, and 11th frequency harmonics, which provide information about the changes in sample mass and softness. First, the bare sensors were measured in air and after NaCl (aqueous) 0.1 M was introduced into the module chamber. The electrolyte resulted in a large shift in the QCM‐D signals due to the change in media density inside the chamber, which was adjusted for when calculating polymer swelling. After acquiring the bare sensor QCM‐D baseline signals in both dry and wet conditions, the sensor was coated with a polymer film and placed back into the analyzer. The QCM‐D signals were recorded again in dry state and with electrolyte after making sure that the frequency, f, and dissipation, D, signals were stabilized (i.e., Δf < 1 Hz per 1 h), typically taking up to several hours, depending on the extent of passive swelling. Using the QSoft software function “stitch data” provided the difference between the f and D signals before and after the sensor was coated with a polymer film in both dry state and with electrolyte. This difference was used to calculate the film areal mass in dry and swollen states, using the Sauerbrey equation:

(3) $$\begin{array}{c} \;\frac{\Delta m}{A}=-\Delta f_{n}\frac{\rho_{q}V_{q}}{2f_{0}^{2}n}\approx \frac{-\Delta f_{n}}{n}17.9\;ng\;cm^{-2}\; \end{array}$$

where Δfn is the frequency shift of the nth overtone, A is the sensor active area, ρq is the density of quartz, Vq is the shear wave velocity in quartz, f0 is the fundamental frequency and n is the overtone number. Film thickness was calculated by dividing the calculated areal mass with film density. In the case where the films no longer retain their rigid characteristics, an increase in quartz oscillation energy loss—dissipation is observed. Increased dissipation indicates that the films have become soft and can no longer be approximated using the Sauerbrey method. The films are then approximated to behave as Kelvin‐Voigt elements, meaning they exhibit viscoelastic properties, and can be described by the equation:

(4) $$\begin{array}{c} \;G^{*}=\mu +2\pi if\eta \; \end{array}$$

where G* is the complex shear modulus, µ is elasticity (kg m^−1^ s^−2^), η is viscosity (kg m^−1^ s^−1^) and f is the frequency. To calculate the mass changes of a thick, viscoelastic film, complex shear modulus was analyzed and fitted using multiple overtones (chosen according to best quality fits). Q‐Tools software was used for the modeling and data analysis. Swelling in Figure S8e was calculated as the percentage change in volume relative to the dry volume:

(5) $$\begin{array}{c} \;swelling_{total}=\frac{Vol_{total}-Vol_{dry}}{Vol_{dry}}\;\times 100\%\; \end{array}$$

eQCM‐D were performed using an Autolab PGstat128N potentiostat coupled with a QSense window electrochemistry module (QWEM 401). The integrated three‐electrode setup comprised an Ag/AgCl reference electrode, Pt counter electrode, and the polymer‐coated Au QSensor acting as the working electrode with an electrochemical active area of 0.7854 cm^2^. The current was recorded using an Autolab PGstat128N potentiostat during doping at various potentials.

## Funding

EU Horizon2020 grant agreement n° 952911, BOOSTER, as well as EPSRC Projects EP/Z536258/1, EP/W017091/1, UKRI2634, and EP/X038777/1. EU Horizon2020 research and innovation programme under the Marie Skłodowska‐Curie grant agreement n° 101212358, COSMIC.

## Conflicts of Interest

The authors declare no conflicts of interest.

## Supporting information




**Supporting File**: adma74978‐sup‐0001‐SuppMat.docx.

## Data Availability

The data that support the findings of this study are available from the corresponding author upon reasonable request.
