# Supplementary material for: Poly(Alkoxythiophene) Rod‐Coil Block Copolymers for Organic Electrochemical Transistors
Source: Adv Mater. 2026 Sep 13;38(61):e74978. doi: 10.1002/adma.74978 (PMC13726706; doi:10.1002/adma.74978)
Supplement: Supplementary file 1 [file ADMA-38-e74978-s001.docx]

Supporting Information

**Poly(alkoxythiophene) rod-coil block copolymers for organic electrochemical transistors**

*Junfu Tian, Wentao Shan, Rachael Found, Paola Mantegazza, Francesco Leoni, Andreas Erhardt, Yuyun Yao, Matus Stredansky, Arianna Magni, Adam Marks, Elena Chulanova, Zeinab Hamid, Yueying Huang, Wanpeng Lu, Xian E Li, Baopu Zhang, Qingpei Wan, Christina Koussef, Matilde Concilio, Ruth Theresia Arwani, Caroline A Ross, Sahika Inal, Giovanni Costantini^*^, Iain McCulloch^*^*

J. Tian, R. Found, A. Erhardt, E. Chulanova, Z. Hamid, Y. Huang, W. Lu, X. E. Li, C. Koussef, M. Concilio, I. McCulloch

Chemistry Research Laboratory, University of Oxford, 12 Mansfield Road, Oxford, OX1 3TA, United Kingdom
e-mail: mailto:iain.mcculloch@chem.ox.ac.uk, iain@princeton.edu, g.costantini@bham.ac.uk

Q. Wan, I. McCulloch

Andlinger Center for Energy and the Environment and Department of Electrical and Computer Engineering, Princeton University, Princeton, NJ, 08544, USA.

W. Shan, S. Inal

Organic Bioelectronics Laboratory, Biological and Environmental Science and Engineering Division, King Abdullah University of Science and Technology (KAUST)Thuwal 23955-6900, Saudi Arabia

P. Mantegazza, F. Leoni, M. Stredansky, G. Costantini

School of Chemistry, University of Birmingham, Edgbaston, Birmingham, B15 2TT, United Kingdom

G. Costantini

School of Physics and Astronomy, University of Birmingham, Edgbaston, Birmingham, B15 2TT, United Kingdom

A. Magni, A. Marks, R. T. Arwani

Department of Materials Science and Engineering, Stanford University, Stanford, California 94305, USA

B. Zhang

Department of Chemical Engineering, Massachusetts Institute of Technology, 77 Massachusetts Avenue, Cambridge, MA 02139, USA

C. A. Ross

Department of Materials Science and Engineering, Massachusetts Institute of Technology, 77 Massachusetts Avenue, Cambridge, MA 02139, USA

**Table of contents**

[Section S1 Experimental procedure and reaction optimization	6](#_Toc237259580)

[1.1 Materials	6](#_Toc237259581)

[1.2 Instruments for general characterizations of chemical products	6](#_Toc237259582)

[1.3 Reaction optimization for synthesis of end-group precise poly(3-alkoxythiophene)	7](#_Toc237259583)

[1.4 Synthetic Procedures	19](#_Toc237259584)

[Preparation of 3-(decyloxy)thiophene	19](#_Toc237259585)

[Preparation of 2,5-dibromo-3-(decyloxy)thiophene	22](#_Toc237259586)

[Preparation of H/Br terminated poly(3-alkoxythiophene)	25](#_Toc237259587)

[Preparation of H/CHO terminated poly(3-alkoxythiophene)	27](#_Toc237259588)

[Preparation of rhodanine-terminated polyethylene glycol or poly(L-lactide)	30](#_Toc237259589)

[Preparation of rhodanine-terminated polyesters	40](#_Toc237259590)

[Rod-coil block coupling	44](#_Toc237259591)

[Section S2 Optoelectronic properties of the block copolymers	56](#_Toc237259592)

[2.1 Photoemission Spectroscopy in Air (PESA)	56](#_Toc237259593)

[2.2 UV-Vis Spectroscopy	58](#_Toc237259594)

[2.3 Cyclic Voltammetry (CV) in acetonitrile electrolyte solution	59](#_Toc237259595)

[Section S3 Electrochemical behaviour of the block copolymers	60](#_Toc237259596)

[3.1 Cyclic Voltammetry (CV) in aqueous electrolyte solution	60](#_Toc237259597)

[3.2 Spectroelectrochemistry (SEC) of selected block copolymer in aqueous electrolyte solution	61](#_Toc237259598)

[Section S4 Scanning Tunnelling Microscopy Imaging	62](#_Toc237259599)

[Section S5 Atomic Force Microscopy (AFM) Imaging	63](#_Toc237259600)

[5.1 High Resolution AFM of P3DOT-*b*-PEG-18	63](#_Toc237259601)

[5.2 AFM phase plots of remaining block copolymers	66](#_Toc237259602)

[Section S6 Organic Electrochemical Transistor Characterization	67](#_Toc237259603)

[6.1 I-V Output and Transfer Curves for as-casted film	67](#_Toc237259604)

[6.2 E-QCMD Plots	69](#_Toc237259605)

[6.3 Chronoamperometry from injected charges	70](#_Toc237259606)

[6.4 Transient response measurement	72](#_Toc237259607)

[Section S7 Grazing Incidence Wide Angle X-ray Scattering (GIWAXS)	75](#_Toc237259608)

[7.1 2-D GIWAXS plots of as-casted block copolymer thin films	75](#_Toc237259609)

[7.2 2-D GIWAXS plots of thermally annealed block copolymer films	76](#_Toc237259610)

[7.3 Out-of-plane Line-cut of block copolymer films	77](#_Toc237259611)

[7.4 In-plane Line-cut of block copolymer films	78](#_Toc237259612)

[7.5 Summary of fitted lamellar and π-π stacking distance	79](#_Toc237259613)

[Section S8 Differential Scanning Calorimetry	80](#_Toc237259614)

[Section S9 Inductively Coupled Plasma Mass Spectrometry Residual Pd Measurement (ICP-MS)	81](#_Toc237259615)

[Section S10 Stability Measurement	82](#_Toc237259616)

[Section S11 References	82](#_Toc237259617)

# Section S1 Experimental procedure and reaction optimization

## 1.1 Materials

Reagents and chemicals were purchased from commercial suppliers, including Sigma-Aldrich, FluoroChem, Alfa Aesar, Acros Organics, Manchester Organics, Insight Biotechnology Ltd, Ambeed Inc and were used as supplied unless stated otherwise. Anhydrous chlorobenzene and tetrahydrofuran were purchased from Sigma Aldrich while all remaining dry solvents were collected from an MBraun SPS800 solvent drying system. P3HT dialdehyde was prepared according to published procedure^[S1]^. Prior to use, all glassware was oven dried at 120^o^C and then cooled to room temperature under reduced pressure. Thin layer chromatography (TLC) was performed on Merck aluminium silica gel 60 F254 precoated plates. Plates were visualized under an ultraviolet lamp (λ_max_ = 254nm or 365nm) and/or by staining with iodine suspended on silica, potassium permanganate solution or phosphomolybdic acid. Column chromatography was carried out on Merck Geduran Silicagel 60 (230 – 400 mesh).

## 1.2 Instruments for general characterizations of chemical products

^1^H and ^13^C nuclear magnetic resonance (*NMR*) spectra were taken on a Bruker AVIII HD 400 or AVIII HD 500 spectrometer. Chemical shifts are quoted in parts per millions (ppm) with multiplicities of the spectra recorded as the following: s, singlet; d, doublet; t, triplet; q, quartet; p, quintet; m, multiplet; br, broad; dd, doublet of doublets et cetera. Coupling constants (J) are given in Hertz (Hz) and rounded to the nearest 0.1 Hz. High resolution ESI mass spectra were recorded on a Bio-Accord LC-MS system, m/z values are reported in Daltons (Da) and high-resolution values are calculated to four decimal places from the molecular formula, with all found values in a tolerance of 5 ppm. MALDI spectra were taken on Autoflex MALDI-TOF/TOF instrument with linear mode unless stated otherwise. Analytical GPC was taken on either (chloroform, via RI-detector and UV-detector with polystyrene standard) was performed on either Agilent 1200 Series GPC-SEC system using HPLC chloroform eluent at 40 ^o^C or Agilent 1260 Infinity II using HPLC chlorobenzene eluent at 80 ^o^C.

## 1.3 Reaction optimization for synthesis of end-group precise poly(3-alkoxythiophene)

**Table S1** Optimization of conditions for the synthesis of end-group precise poly(3-alkoxythiophene)

| Entry | Catalyst | Condition | M_n_/kDa | PDI | Yield |
| --- | --- | --- | --- | --- | --- |
| 1 | Ni(dppp)Cl_2_ | rt, 30 mins | 7.0 | 1.4 | 29% |
| 2 |  | reflux, 30 mins | 10.7 | 1.5 | 33% |
| 3 |  | reflux, overnight | 9.4 (D = 1.3), 57.6 (1.1) | Bimodal distribution | 42% |
| 4 |  | rt, 30 mins, end capped with ethynyl magnesium bromide | 10.8 | 1.4 | 35% |
| 5 | Ni(dppf)Cl2 | rt, 30 mins | 4.2 | 1.5 | 18% |
| 6 | Pd-PEPPSi-IPr | rt, 30 mins | 6.4 | 1.4 | 33% |
| 7 |  | rt, overnight | 7.3 | 1.4 | 31% |
| 8 | Ni(IPr)(PPh_3_)Cl_2_ | rt, 30 mins | 15.9 | 1.4 | 74% |

**Figure S1** GPC elution curve and MALDI-TOF spectra of samples from Condition 1

**Figure S2** GPC elution curve and MALDI-TOF spectra of samples from Condition 2

**Figure S3** GPC elution curve and MALDI-TOF spectra of samples from Condition 3

**Figure S4** GPC elution curve and MALDI-TOF spectra of samples from Condition 4

**Figure S5** GPC elution curve and MALDI-TOF spectra of samples from Condition 5

**Figure S6** GPC elution curve and MALDI-TOF spectra of samples from Condition 6

**Figure S7** GPC elution curve and MALDI-TOF spectra of samples from Condition 7

**Figure S8** GPC elution curve and MALDI-TOF spectra of samples from Condition 8

**Table S2** Optimization of condition for the coupling of different coil blocks to P3HT dialdehyde. (a) 6 eq of PEG-2k coupling block was used (b) 12 eq of PCL-2k and PLLA-2k coupling blocks were used.

| Entry | Coil block | Reaction conditions | End-group conversion (%) | Coil block stability |
| --- | --- | --- | --- | --- |
| 1 | PEG^a^ | NEt_3_, CHCl_3_, reflux, 24 h | 36 | ✔ |
| 2 |  | NEt_3_, PhCl, 90 ^o^C, 24 h | 51 | ✔ |
| 3 |  | Piperidine, CHCl_3_, reflux, 24 h | 100 | ✔ |
| 4 | PCL^b^ |  |  | ✔ |
| 5 | PLLA^b^ |  | 100 | ❌ |
| 6 |  | DIPEA, CHCl_3_, reflux, 24 h | 27 | ✔ |
| 7 |  | Pyridine, CHCl_3,_ reflux, 24 h | 0 | ✔ |
| 8 |  | Morpholine, CHCl_3,_ reflux, 24 h | 0 | ❌ |
| 9 |  | DBU, CHCl_3_, reflux, 24 h | 100 | ❌ |
| 10 |  | 2,2,6,6-tetramethylpiperidine, CHCl_3,_ reflux, 24 h | 85 | ✔ |
| 11 |  | 2,2,6,6-tetramethylpiperidine, CHCl_3_, reflux, 48 h | 100 | ✔ |

**(a)**

**(b)**

**Figure S9** (a) 1H NMR of polymer (b) GPC trace of polymer sample before and after Knoevenagel coupling

(a) **(b)**

**Figure S10** Plot of retention time of (a) P3DOT-b-PEG series, (b) P3DOT-b-PLLA series

## 1.4 Synthetic Procedures

#### Preparation of 3-(decyloxy)thiophene

Under N_2_, 3-methoxythiophene (4.00 g, 35.0 mmol, 1 eq) was dissolved in toluene (40 mL). Then, 1-decanol (8.31 g, 10.0 mL, 52.5 mmol, 1.5 eq), followed by p-toluenesulfonic acid monohydrate (603 mg, 3.50 mmol, 0.1 eq), was added and the reaction mixture was heated to reflux overnight. Upon completion of reaction, the reaction mixture was diluted with diethyl ether (160 mL) and washed with saturated sodium bicarbonate solution (150 mL), water (150 mL x 2) and dried over anhydrous sodium sulphate. The crude product was further purified by column chromatography (petroleum ether) to yield the product as a colourless oil. (6.90 g, 82%). ^1^H NMR (400 MHz, CDCl3) δ 7.17 (dd, J = 5.2, 3.1 Hz, 1H, Ar H), 6.76 (dd, J = 5.2, 1.5 Hz, 1H, Ar H), 6.23 (dd, J = 3.1, 1.5 Hz, 1H, Ar H), 3.94 (t, J = 6.6 Hz, 2H, CH_2_), 1.88 – 1.67 (m, 2H, CH_2_), 1.52 – 1.40 (m, 2H, CH_2_), 1.40 – 1.19 (m, 12H, CH_2_), 0.96 – 0.81 (m, 3H, CH_3_). ^13^C NMR (101 MHz, CDCl_3_) δ 158.20, 124.62, 119.68, 97.09, 70.42, 32.04, 29.72, 29.70, 29.54, 29.46, 29.42, 26.20, 22.82, 14.25. HRMS (ESI) *m/z*: [M]^+^ calcd for C_14_H_24_OS, 240.1542; found, 240.1544 (0.7 ppm).

**Figure S11** ^1^H, ^13^C NMR and HRMS of 3-(decyloxy)thiophene

#### Preparation of 2,5-dibromo-3-(decyloxy)thiophene

Under N_2_, 3-(docyloxy)thiophene (2.20 g, 9.15 mmol, 1 eq) was dissolved in anhydrous THF (20 mL) and was cooled to 0 ^o^C. Then, N-Bromosuccinimide (3.58 g, 20.13 mmol, 2.2 eq) was added. The reaction mixture was protected from light with aluminium foil and left to stir at room temperature for 3 hours. Upon completion of reaction, the reaction mixture was diluted with diethyl ether (180 mL) and washed with saturated sodium bicarbonate solution (150 mL), water (150 mL x 2) and dried over anhydrous sodium sulphate. The crude product was further purified by column chromatography (petroleum ether) to yield the product as a colourless oil. (2.80 g, 77%). ^1^H NMR (400 MHz, CDCl_3_) δ 6.76 (s, 1H, Ar H), 3.99 (t, *J* = 6.6 Hz, 2H, CH_2_), 1.73 (dq, *J* = 8.2, 6.6 Hz, 2H, CH_2_), 1.44 (td, *J* = 8.1, 4.9 Hz, 2H, CH_2_), 1.42 – 1.25 (m, 12H, CH_2_), 0.92 – 0.84 (m, 3H, CH_3_). ^13^C NMR (101 MHz, CDCl_3_) δ 153.99, 121.02, 109.73, 90.68, 72.74, 32.04, 29.68, 29.53, 29.46, 29.43, 25.90, 22.83, 14.27. HRMS (ESI) *m/z*: [M + H]^+^ calcd for C_14_H_23_Br_2_OS, 396.9831; found 396.9835 (1.0 ppm).

**Figure S12** ^1^H, ^13^C NMR and HRMS of 2,5-dibromo-3-(decyloxy)thiophene

#### Preparation of H/Br terminated poly(3-alkoxythiophene)

Under N_2_, 2,5-dibromo-3-(decyloxy) thiophene (1820 mg, 4.57 mmol) was dissolved in anhydrous THF (45.7 mL). Then, the solution was cooled to 0 ^o^C and ^i^PrMgCl (2.06 mL, 2.0M, 0.9 eq) was added. The reaction mixture was cooled to 0 ^o^C for 10 minutes, before being left at room temperature for another 50 minutes. After which, an anhydrous solution of Ni(IPr)(PPh_3_)Cl_2_ (2.00 mL, 20 mg/mL in THF) was added. The reaction mixture was stirred at room temperature for 30 minutes, before being precipitated in methanol. The crude polymer was filtered and further purified by Soxhlet extraction in acetone (4 hours), hexane (4 hours) and finally extracted in chloroform. The chloroform fraction was concentrated in vacuo and precipitated once more in methanol to yield a dark blue solid (825 mg, 74%). ^1^H NMR (400 MHz, CDCl_3_) ^1^H NMR (400 MHz, CDCl_3_) δ 6.92 (s, 1H), 4.16 (s, 2H), 1.89 (s, 2H), 1.59 (s, 2H), 1.27 (s, 12H), 0.86 (s, 3H). GPC (PhCl): M_n_ = 15.9 kDa, M_w_ = 20.9 kDa.

**Figure S13** ^1^H, and GPC plots of H/Br-terminated P3DOT

#### Preparation of H/CHO terminated poly(3-alkoxythiophene)

*Reaction and workup* P3DOT H/Br (800 mg, 0.053 mmol), (4-formylphenyl) boronic acid (160 mg, 1.06 mmol, 20 eq), Pd-G_3_-P^t^Bu_3_ (45.76 mg, 0.080 mmol, 1.5 eq) and anhydrous potassium phosphate (900 mg, 4.24 mmol, 80 eq) were dissolved in degassed THF (80 mL) and water (1.60 mL). The reaction mixture was warmed to reflux overnight, then precipitated and further purified by Soxhlet extraction (methanol -> acetone -> chloroform). The extractant from chloroform is concentrated in vacuo to about 150 mL in volume.

*Palladium scavenging* Sodium diethyldithiocarbamate (1.50 g) was dissolved in water (150 mL) and added to the chloroform extractant. Then, the biphasic mixture was stirred at reflux for 24 hours and cooled to room temperature. The layers were left to separate and the organic layer was washed with water (150 mL x 2). Then, more solution of sodium diethyldithiocarbamate (1.50 g) in water (150 mL) was added to the chloroform extractant and the Pd scavenging process was repeated for another two times, before the cleaned organic layer was finally concentrated and precipitated in methanol, then purified further by Soxhlet extraction with methanol overnight to remove sodium diethyldithiocarbamate remaining in the organic phase. The product was finally extracted with chloroform, concentrated and precipitated in methanol to yield the purified P3DOT H/CHO solid (783 mg, 98%). ^1^H NMR (400 MHz, CDCl_3_) δ 9.84 (t, *J* = 6.9 Hz, 1H, CHO H), 6.91 (s, 53H, Ar H), 4.17 (s, 128H, CH_2_), 1.89 (d, *J* = 13.0 Hz, 123H, CH_2_), 1.54 – 1.26 (m, 768H, CH_2_), 0.87 (dt, *J* = 7.2, 3.5 Hz, 163H, CH_3_).

**Figure S14** ^1^H, MALDI-TOF-MS and GPC plots of H/CHO-terminated P3DOT

#### Preparation of rhodanine-terminated polyethylene glycol or poly(L-lactide)

Under N_2,_ alcohol-terminated PEG or PLLA (1.0 eq) and 2-(4-oxo-2-thioxothiazolidin-3-yl)acetic acid (2.0 eq) were dissolved in anhydrous DCM (to a concentration of 100 mg/mL w.r.t. coil precursor). Then, DMAP (0.2 eq), followed by DCC (2.0 eq), was added and the reaction mixture was stirred at room temperature for 16 hours. Then, ethanol (2.0 eq) was added to convert any unreacted acid to the corresponding ester and DCC to DCU respectively. The reaction mixture was then diluted with acetonitrile 10 times the volume, cooled to 0 ^o^C for 1 hour, filtered and washed with additional cold acetonitrile, then dried in vacuo. This process was repeated 2 – 3 times until no visible precipitation of DCU was following the solvent removal. Then, the crude polymer was further purified by column chromatography.

Note: Polymer of around 5-6 kDa is around the highest molecular weight polymer which can pass through silica column chromatography.

*Example Procedure: Preparation of PEG-Rh-4k*

Polyethylene glycol monomethyl ether (5k, commercially available, a mixture of 4 kDa and 8 kDa) (1875 mg, 0.38 mmol), 2-(4-oxo-2-thioxothiazolidin-3-yl)acetic acid (107 mg, 0.75 mmol) were dissolved in anhydrous DCM (19 mL). Then, DMAP (9.15 mg, 0.075 mmol), followed by DCC (116 mg, 0.75 mmol), was added and the reaction mixture was stirred overnight. Completion of reaction was monitored by MALDI-TOF-MS. The product was diluted with acetonitrile, then repeatedly filtered, concentrated, diluted as described in General Procedure above. The product was further purified by column chromatography (chloroform/methanol 19/1 -> chloroform/methanol 85/15) to yield a white solid (823 mg, 44%). ^1^H NMR (400 MHz, CDCl_3_) δ 4.76 (s, 2H, CH_2_), 4.31 (t, *J* = 4.7 Hz, 2H, CH_2_), 4.11 (s, 2H, CH_2_), 3.64 (s, 342H, CH_2_), 3.46 (t, *J* = 5.0 Hz, 2H, CH_2_), 3.38 (d, *J* = 1.1 Hz, 3H, CH_3_). GPC (CHCl_3_): M_n_ = 5.5 kDa, M_w_ = 7.3 kDa

Note: Increasing the eluent ratio beyond chloroform/methanol 4/1 starts to elute PEG-Rh-8k.

**Figure S15** Differences in MALDI-TOF-MS before and after anchoring rhodanine terminus

**Figure S16** ^1^H, MALDI-TOF-MS and GPC plots of PEG-Rh-4k

*Preparation of PEG-Rh-2k* Polyethylene glycol monomethyl ether (2k, commercially available) (1500 mg, 0.75 mmol), 2-(4-oxo-2-thioxothiazolidin-3-yl)acetic acid (215 mg, 1.50 mmol), DMAP (18.3 mg, 0.15 mmol), DCC (232 mg, 1.50 mmol) and DCM (17 mL) were used. The crude product was purified by column chromatography (chloroform/ethanol 39/1 -> chloroform/ethanol 37/3) to yield the product as a white solid. (1159 mg, 77%). ^1^H NMR (400 MHz, CDCl_3_) δ 4.76 (s, 2H, CH_2_), 4.34 – 4.28 (m, 2H, CH_2_), 4.11 (s, 2H, CH_2_), 3.85 – 3.77 (m, 1H, CH_2_), 3.64 (s, 188H, CH_2_), 3.50 – 3.43 (m, 1H, CH_2_), 3.38 (s, 3H, CH_3_). GPC (CHCl_3_): M_n_ = 2.1 kDa, M_w_ = 2.7 kDa

**Figure S17** ^1^H, MALDI-TOF-MS and GPC plots of PEG-Rh-2k

*Preparation of PEG-Rh-750* Polyethylene glycol monomethyl ether (750, commercially available) (1500 mg, 2.00 mmol), 2-(4-oxo-2-thioxothiazolidin-3-yl)acetic acid (764 mg, 4.00 mmol), DMAP (48.9 mg, 0.40 mmol), DCC (825 mg, 4.00 mmol) and DCM (23 mL) were used. The crude product was purified by column chromatography (chloroform -> chloroform/ethanol 24/1) to yield the product as a white solid. (1159 mg, 77%). ^1^H NMR (400 MHz, CDCl_3_) δ 4.69 (s, 2H, CH_2_), 4.25 (dd, J = 5.8, 3.6 Hz, 2H, CH_2_), 4.04 (s, 2H, CH_2_), 3.81 – 3.37 (m, 68H, CH_2_), 3.31 (s, 3H, CH_3_). GPC (CHCl_3_): M_n_ = 706 Da M_w_ = 866 Da

**Figure S18** ^1^H, MALDI-TOF-MS and GPC plots of PEG-Rh-750

*Preparation of PEG-Rh-450* Polyethylene glycol monomethyl ether (450, commercially available) (1200 mg, 2.54 mmol), 2-(4-oxo-2-thioxothiazolidin-3-yl)acetic acid (972 mg, 5.08 mmol), DMAP (62.0 mg, 0.51 mmol), DCC (1152 mg, 5.08 mmol) and DCM (19 mL) were used. The crude product was purified by column chromatography (chloroform -> chloroform/methanol 49/1) to yield the product as an orange oil. (1019 mg, 85%). ^1^H NMR (400 MHz, CDCl3) δ 4.75 (s, 2H, CH_2_), 4.34 – 4.26 (m, 2H, CH_2_), 4.10 (s, 2H, CH_2_), 3.85 – 3.48 (m, 38H, CH_2_), 3.37 (s, 3H, CH_3_). GPC (CHCl_3_): M_n_ = 525 Da, M_w_ =584 Da

**Figure S19** ^1^H and GPC plots of PEG-Rh-450

#### Preparation of rhodanine-terminated polyesters

Benzyl-substituted PLLA were prepared similar to published procedure with Sn(Oct)_2_ as a Lewis acid^[2,3]^. Upon completion, the reaction mixture was diluted with chloroform, then washed with saturated sodium bicarbonate solution, water, and then dried in vacuo, before being precipitated in cold diethyl ether to yield the rhodanine-terminated polyesters.

*Preparation of PLLA-Rh-5k* α-benzyl-ω-hydroxy-poly(L-lactide) (5k) (828 mg, 0.20 mmol), 2-(4-oxo-2-thioxothiazolidin-3-yl)acetic acid (95.5 mg, 0.50 mmol), DMAP (6.10 mg, 0.050 mmol), EDCI (86.9 mg, 0.56 mmol) and anhydrous DCM (16 mL) were used, yielding PLLA-Rh-5k as a white solid. (655 mg, 79%). ^1^H NMR (400 MHz, CDCl_3_) δ 7.34 (td, *J* = 7.5, 5.2 Hz, 5H, Ar H), 5.16 (q, *J* = 7.1 Hz, 66H, CH), 4.87 (s, 1H, CH_2_), 4.78 (s, 1H, CH_2_), 4.07 (s, 2H, CH_2_), 1.58 (d, *J* = 7.1 Hz, 224H, CH_3_). GPC (CHCl_3_): M_n_ = 4.8 kDa, M_w_ =7.0 kDa

**Figure S20** ^1^H and GPC plots of PLLA-Rh-5k

*Preparation of PLLA-Rh-2k* α-benzyl-ω-hydroxy-poly(L-lactide) (2k) (1012 mg, 0.40 mmol), 2-(4-oxo-2-thioxothiazolidin-3-yl)acetic acid (191 mg, 1.00 mmol), DMAP (12.20 mg, 0.10 mmol), EDCI (173 mg, 1.12 mmol) and anhydrous DCM (24 mL) were used, yielding PLLA-Rh-2k as a white solid. (687 mg, 68%). ^1^H NMR (400 MHz, CDCl_3_) δ 7.34 (td, *J* = 7.7, 5.3 Hz, 5H, Ar H), 5.16 (q, *J* = 7.1 Hz, 37H, CH), 4.89 (d, *J* = 17.0 Hz, 1H, CH_2_), 4.75 (d, *J* = 16.9 Hz, 1H, CH_2_), 4.07 (s, 2H, CH_2_), 1.58 (d, *J* = 7.1 Hz, 114H, CH_3_). GPC (CHCl_3_): M_n_ = 2.8 kDa M_w_ =3.8 kDa

**Figure S21** ^1^H and GPC plots of PLLA-Rh-3k

#### Rod-coil block coupling

*Example Procedure: Preparation of P3DOT-b-PEG-10*

Under N_2_, P3DOT H/CHO (30 mg, 15.0 kDa, 0.002 mmol) and PEG-10-Rh (7.7 mg, 0.012 mmol) were added to a microwave vial. Then, anhydrous CHCl_3_ (4.5 mL) and piperidine (0.060 mL, 0.60 mmol, 50 eq w.r.t. PEG) were added and the reaction mixture was warmed to reflux for 24 hours. Then, the reaction mixture was precipitated in methanol and further purified by Soxhlet extraction with acetonitrile, then chloroform. The chloroform layer was washed twice with water and concentrated, then re-precipitated in methanol to yield a dark blue solid (29 mg). ^1^H NMR (400 MHz, CDCl_3_) δ 6.92 (s, 30H, Ar H), 4.16 (s, 80H, CH_2_), 3.65 (d, *J* = 4.0 Hz, 38H, CH_2_), 3.38 (s, 4H, CH_3_), 1.90 (s, 105H, CH_2_), 1.57 – 1.12 (m, 776H, CH_2_), 0.87 (tt, *J* = 7.0, 3.1 Hz, 221H, CH_3_). GPC (PhCl): M_n_ = 16.3 kDa, M_w_ = 20.1 kDa

**Figure S22** ^1^H and GPC plots of P3DOT-b-PEG-10

*Preparation of P3DOT-b-PEG-18* P3DOT H/CHO (30 mg, 0.002 mmol), PEG-18-Rh (12 mg, 0.012 mmol), anhydrous CHCl_3_ (4.5 mL) and piperidine (0.060 mL) were used, yielding a dark blue solid (30 mg). ^1^H NMR (400 MHz, CDCl_3_) δ 6.92 (s, 43H, Ar H), 4.16 (s, 99H, CH_2_), 3.65 (d, *J* = 4.9 Hz, 68H, CH_2_), 3.38 (s, 5H, CH_3_), 2.06 – 1.75 (m, 126H, CH_2_), 1.56 – 1.08 (m, 909H, CH_2_), 0.87 (dd, *J* = 8.3, 5.0 Hz, 192H, CH_3_). GPC (PhCl): M_n_ =16.5 kDa, M_w_ = 25.0 kDa

**Figure S23** ^1^H and GPC plots of P3DOT-b-PEG-18

*Preparation of P3DOT-b-PEG-40* P3DOT H/CHO (30 mg, 0.002 mmol), PEG-40-Rh (26 mg, 0.012 mmol), anhydrous CHCl_3_ (4.5 mL) and piperidine (0.060 mL) were used, yielding a dark blue solid (29 mg). ^1^H NMR (400 MHz, CDCl_3_) δ 6.95 (s, 47H, Ar H), 4.17 (s, 113H, CH_2_), 3.64 (s, 188H, CH_2_), 3.38 (s, 3H, CH_3_), 2.02 – 1.77 (m, 146H, CH_2_), 1.61 – 1.19 (m, 1087H, CH_2_), 0.98 – 0.73 (m, 235H, CH_3_). GPC (PhCl): M_n_ = 17.4 kDa, M_w_ = 25.2 kDa

**Figure S24** ^1^H and GPC plots of P3DOT-b-PEG-40

*Preparation of P3DOT-53-b-PEG-80* P3DOT H/CHO (30 mg, 0.002 mmol), PEG-80-Rh (52 mg, 0.012 mmol), anhydrous CHCl_3_ (4.5 mL) and piperidine (0.060 mL) were used, yielding a dark blue solid (32 mg). ^1^H NMR (400 MHz, CDCl_3_) δ 7.12 – 6.69 (m, 45H, Ar H), 4.45 – 3.96 (m, 104H, CH_2_), 3.64 (s, 382H, CH_2_), 3.47 – 3.33 (m, 4H, CH_3_), 1.91 (dd, *J* = 14.2, 6.7 Hz, 124H, CH_2_), 1.62 – 1.13 (m, 1007H, CH_2_), 0.87 (td, *J* = 6.8, 2.9 Hz, 215H, CH_3_). GPC (PhCl): M_n_ = 19.5 kDa, M_w_ = 26.4 kDa

**Figure S25** ^1^H and GPC plots of P3DOT-b-PEG-80

*Preparation of P3DOT-53-b-PLLA-34* P3DOT H/CHO (30 mg, 15.0 kDa, 0.002 mmol) and PLLA-34-Rh (44 mg, 0.016 mmol) were added to a microwave vial. Then, anhydrous CHCl_3_ (4.5 mL) and 2,2,6,6-tetramethylpiperidine (0.14 mL, 0.8 mmol, 50 eq w.r.t. PLLA) were added and the reaction mixture was warmed to reflux for 36 hours. Then, the reaction mixture was precipitated in methanol and further purified by Soxhlet extraction with acetonitrile, then chloroform, which was concentrated and re-precipitated in methanol to yield a dark blue solid (33 mg). ^1^H NMR (400 MHz, CDCl_3_) δ 6.91 (s, 32H, Ar H), 5.16 (q, *J* = 7.1 Hz, 36H, CH), 4.16 (s, 84H, CH_2_), 1.90 (s, 111H, CH_2_), 1.76 – 1.47 (m, 651H), 1.47 – 1.06 (m, 1294H), 0.89 (td, *J* = 17.1, 6.7 Hz, 413H). GPC (PhCl): M_n_ = 16.5 kDa M_w_ = 24.1 kDa

**Figure S26** ^1^H and GPC plots of P3DOT-b-PLLA-34

*Preparation of P3DOT-53-b-PLLA-65* P3DOT H/CHO (30 mg, 0.002 mmol), PLLA-65-Rh (79 mg, 0.016 mmol), anhydrous CHCl_3_ (4.5 mL) and 2,2,6,6-tetramethylpiperidine (0.14 mL) were used, yielding a dark blue solid (33 mg). ^1^H NMR (400 MHz, CDCl_3_) δ 7.40 – 7.30 (m, 6H, Ar H), 6.91 (s, 30H, Ar H), 5.16 (q, *J* = 7.1 Hz, 66H, CH), 4.16 (s, 90H, CH_2_), 1.90 (s, 214H), 1.56 (s, 835H), 1.33 (d, *J* = 51.5 Hz, 1569H), 1.05 – 0.75 (m, 494H). GPC (PhCl): M_n_ = 18.3 kDa, M_w_ = 25.9 kDa.

**Figure S27** ^1^H and GPC plots of P3DOT-b-PLLA-65

# Section S2 Optoelectronic properties of the block copolymers

This section outlines the frontier energy levels of all the block copolymers. A summarized plot of the highest occupied molecular orbital (HOMO) and lowest unoccupied molecular orbital (LUMO) is provided in Figure S27. Notably, the HOMO was determined by photoemission spectroscopy in air (PESA) (Figure S28) and compared with estimates based on cyclic voltammetry (CV) in acetonitrile using tetrabutylammonium phosphorous pentafluoride (TBAPF_6_) as electrolyte (Figure S30). The optical band gap of all samples was determined from the onset energy in UV-Vis spectroscopy (Figure S29), showing that all block copolymers with the same rod backbone share an identical optical band gap. The LUMO levels of the materials were then further evaluated using the HOMO (from PESA) and the measured optical band gap.

## 2.1 Photoemission Spectroscopy in Air (PESA)

**Figure S28** Summary of HOMO and LUMO of all polymer samples with respect to vacuum level

**(a)**

**(b)**

**(c)**

**(d)**

**(e)**

**(f)**

**(g)**

**Figure S29** PESA plots of (a) P3DOT; (b) P3DOT-b-PEG-10; (c) P3DOT-b-PEG-18; (d) P3DOT-b-PEG-40; (e) P3DOT-b-PEG-80; (f) P3DOT-b-PLLA-34; (g) P3DOT-b-PLLA-65

## 2.2 UV-Vis Spectroscopy

**Figure S30** UV-Vis spectra of all polymer samples

## 2.3 Cyclic Voltammetry (CV) in acetonitrile electrolyte solution

**(a)**

**(b)**

**(c)**

**(d)**

**(e)**

**(f)**

**(g)**

**Figure S31** Cyclic voltammograms of all polymer samples in acetonitrile with 0.1 M tetrabutylammonium hexafluorophosphate as supporting electrolyte: (a) P3DOT, (b) P3DOT-b-PEG-10, (c) P3DOT-b-PEG-18, (d) P3DOT-b-PEG-40, (e) P3DOT-b-PEG-80, (f) P3DOT-b-PLLA-34, (g) P3DOT-b-PLLA-65

# Section S3 Electrochemical behaviour of the block copolymers

## 3.1 Cyclic Voltammetry (CV) in aqueous electrolyte solution

**(a)**

**(b)**

**(c)**

**(d)**

**(e)**

**(f)**

**(g)**

**Figure S32** Cyclic voltammograms of all polymer samples in water with 0.1 M sodium chloride as supporting electrolyte: (a) P3DOT, (b) P3DOT-b-PEG-10, (c) P3DOT-b-PEG-18, (d) P3DOT-b-PEG-40, (e) P3DOT-b-PEG-80, (f) P3DOT-b-PLLA-34, (g) P3DOT-b-PLLA-65

## 3.2 Spectroelectrochemistry (SEC) of selected block copolymer in aqueous electrolyte solution

**Figure S33** SEC plots of P3DOT-b-PEG

# Section S4 Scanning Tunnelling Microscopy Imaging

**Figure S34** STM image of P3DOT-b-PEG-18 adsorbed on Au(111). The inset schematically illustrates the relative orientation of the polymer side chains (thin line) with respect to the backbone (thick line), as adopted by the same polymer in 3D thin films. This simplified structural model is superposed onto selected regions of the STM image to demonstrate the good (red) and poor (dark blue) agreement with the experimentally observed 2D conformation.

Figure S34 shows an isolated P3DOT-b-PEG-18 molecule adsorbed on Au(111), with its PEG terminus on the top left of the image. Individual OC_10_H_21_ side chains are clearly resolved as straight, elongated structures extending form the central polythiophene backbone. While some of them form an angle of approximately 30°–35° with respect to the backbone, others adopt a more perpendicular orientation, resulting in an increased overall molecular width.

# Section S5 Atomic Force Microscopy (AFM) Imaging

# 5.1 High Resolution AFM of P3DOT-*b*-PEG-18

**Figure S35** Large-scale AFM height images of the surface of P3DOT-*b*-PEG-18 thin films. (a) Non-annealed sample and (b) annealed sample. The insets display selected regions at enhanced contrast to highlight morphological details. Height profiles extracted along the lines marked in the insets are shown below the corresponding images. The images were acquired using the first eigenmode of the Multi75-Al-G probe, oscillating at a resonance frequency of approximately 80 kHz, with a scan rate of 2.4 Hz and a resolution of 512 × 512 pixels.

Figure S35 presents a direct comparison of the surface morphology of P3DOT-*b*-PEG-18 thin films before and after thermal annealing to 150 °C for 30 min under a nitrogen atmosphere. The non-annealed film (Figure S35a) appears morphologically homogeneous, exhibiting low surface roughness with shallow height variations of approximately 1–5 nm. In contrast, annealing (Figure S35b) leads to the formation of pronounced mounds and pits with lateral dimensions of ~50-100 nm and vertical heights of approximately 10-20 nm. This substantial modification of the surface morphology is consistent with the onset of phase separation, which may, in turn, contribute to the reduced device efficiency observed following thermal annealing.

**Figure S36** High-resolution AFM phase images of the surface of P3DOT-53-b-PEG-18 thin films. (a) non-annealed sample and (b) annealed sample. Images were acquired using the second eigenmode of the Multi75-Al-G probe, oscillating at a resonance frequency of 531 kHz, with a scan rate of 5.1 Hz and a resolution of 1024 × 1024 pixels.

Figure S36a shows a high-resolution AFM image of the surface of a non-annealed P3DOT-53-b-PEG-18 thin film, revealing the presence of short, locally parallel but overall slightly undulating features. These extend over lengths of approximately 40–60 nm and exhibit characteristic separations of about 3–4 nm, dimensions comparable to the single-molecule scale. These features are therefore assigned to individual polymer chains lying parallel to each other and adopting a predominantly face-on orientation at the film surface. Notably, the observed interchain separations closely match those measured by ESD–STM at monolayer coverage (Figure 2c), as further illustrated by a direct comparison of AFM and STM images scaled to the same dimensions in Figure S36.

Although these AFM observations appear to contrast with GIWAXS measurements, which indicate a preferential edge-on orientation of the polymer chains (Figure 3), it should be noted that AFM probes only the surface of the film, whereas GIWAXS provides a bulk-averaged structural description. A plausible interpretation of both datasets is that the polymer chains adopt a predominantly edge-on orientation in the bulk of the thin film, while favouring a face-on orientation at the surface. This surface–bulk structural disparity likely reflects the different energetic constraints at the film–air interface, where reduced intermolecular coordination alters the balance of packing interactions.

Interestingly, despite the increased surface roughness observed after thermal annealing, similar parallel features are also detected on annealed films (Figure S36b), indicating that the face-on surface morphology is preserved upon annealing.

**Figure S37** High-resolution AFM phase image of the surface of a P3DOT-53-b-PEG-18 thin film. The inset shows two juxtaposed STM images of an ultrathin film of the same polymer deposited by ESD on Au(111). The AFM and STM images are scaled to the same dimensions, enabling a direct comparison that highlights the similarity between the spacing of the parallel features observed by AFM and the separation between individual face-on polymer chains resolved by STM. The AFM image was acquired using the second eigenmode of the Multi75-Al-G probe, oscillating at a resonance frequency of 531 kHz, with a scan rate of 10 Hz and a resolution of 1024 × 1024 pixels.

## 5.2 AFM phase plots of remaining block copolymers

**Figure S38** AFM phase plot of remaining polymer samples: (a) P3DOT, (b) P3DOT-b-PEG-10, (c) P3DOT-b-PEG-40, (d) P3DOT-b-PEG-80, (e) P3DOT-b-PLLA-34, (f) P3DOT-b-PLLA-65

# Section S6 Organic Electrochemical Transistor Characterization

## 6.1 I-V Output and Transfer Curves for as-casted film

**P3DOT-b-PEG-10**

**P3DOT-b-PEG-18**

**P3DOT-b-PEG-40**

**P3DOT-b-PEG-80**

**P3DOT-b-PLLA-34**

**P3DOT-b-PLLA-65**

**(a)**

**(b)**

**(c)**

**(d)**

**(e)**

**(f)**

**(g)**

**Figure S39** Output of all as-casted block copolymers: (a) P3DOT, (b) P3DOT-b-PEG-10, (c) P3DOT-b-PEG-18, (d) P3DOT-b-PEG-40, (e) P3DOT-b-PEG-80, (f) P3DOT-b-PLLA-34, (g) P3DOT-b-PLLA-65

**Figure S40** Transfer curve of all as-casted block copolymers: (a) P3DOT, (b) P3DOT-b-PEG-10, (c) P3DOT-b-PEG-18, (d) P3DOT-b-PEG-40, (e) P3DOT-b-PEG-80, (f) P3DOT-b-PLLA-34, (g) P3DOT-b-PLLA-65

6.2 E-QCMD Plots

**Figure S41** E-QCMD plots of all polymer samples: (a) P3DOT, (b) P3DOT-b-PEG-10, (c) P3DOT-b-PEG-18, (d) P3DOT-b-PEG-40, (e) P3DOT-b-PEG-80, (f) P3DOT-b-PLLA-34, (g) P3DOT-b-PLLA-65

6.3 Chronoamperometry from injected charges

**Figure S42** Plots of injected charge density against applied voltage. Volumetric capacitance was extracted from the slope of the graph. (a) P3DOT, (b) P3DOT-b-PEG-10, (c) P3DOT-b-PEG-18, (d) P3DOT-b-PEG-40, (e) P3DOT-b-PEG-80, (f) P3DOT-b-PLLA-34, (g) P3DOT-b-PLLA-65

**Figure S43** Summary of volumetric capacitance determined from chronoamperometry of injected charges.

## 6.4 Transient response measurement

**Figure S44** Plot of normalized IDS against time. The device was biased at t = 10 s and the current response curve was fitted to obtain τon: (a) P3DOT, (b) P3DOT-b-PEG-10, (c) P3DOT-b-PEG-18, (d) P3DOT-b-PEG-40, (e) P3DOT-b-PEG-80, (f) P3DOT-b-PLLA-34, (g) P3DOT-b-PLLA-65

**Figure S45** Plot of normalized IDS against time. The device was unbiased at t = 20 s and the current response curve was fitted to obtain τoff: (a) P3DOT, (b) P3DOT-b-PEG-10, (c) P3DOT-b-PEG-18, (d) P3DOT-b-PEG-40, (e) P3DOT-b-PEG-80, (f) P3DOT-b-PLLA-34, (g) P3DOT-b-PLLA-65

**Table S3** Parameters obtained from bi-exponential fitting of the OECT turn-on transients shown in Figure S43. tauon is the amplitude-weighted average, tau_on_ = (A_1_*τ_1,on_ + A_2_*τ_2,on_)/(A_1_ + A_2_).

| **Polymer** | **A_1_** | **τ_1,on_ / s** | **A_2_** | **τ_2,on_ / s** | **A_1_/(A_1_+A_2_)** | **τ_on_ / s** |
| --- | --- | --- | --- | --- | --- | --- |
| P3DOT | 0.708 | 0.472 | 0.543 | 7.35 | 0.566 | 3.46 |
| P3DOT-b-PEG-10 | 0.514 | 0.625 | 0.550 | 4.59 | 0.483 | 2.68 |
| P3DOT-b-PEG-18 | 0.809 | 0.453 | 0.488 | 3.87 | 0.624 | 1.74 |
| P3DOT-b-PEG-40 | 0.888 | 0.348 | 0.290 | 4.06 | 0.754 | 1.26 |
| P3DOT-b-PEG-80 | 1.732 | 0.116 | 0.303 | 4.01 | 0.851 | 0.70 |
| P3DOT-b-PLLA-33 | 0.794 | 0.475 | 0.378 | 8.10 | 0.677 | 2.94 |
| P3DOT-b-PLLA-65 | 1.192 | 0.384 | 0.431 | 5.29 | 0.734 | 1.69 |

# Section S7 Grazing Incidence Wide Angle X-ray Scattering (GIWAXS)

## 7.1 2-D GIWAXS plots of as-casted block copolymer thin films

**Figure S46** 2-D GIWAXS plots of as-casted block copolymer films: (a) P3DOT, (b) P3DOT-b-PEG-10, (c) P3DOT-b-PEG-18, (d) P3DOT-b-PEG-40, (e) P3DOT-b-PEG-80, (f) P3DOT-b-PLLA-34, (g) P3DOT-b-PLLA-65

## 7.2 2-D GIWAXS plots of thermally annealed block copolymer films

**Figure S47** 2-D GIWAXS plots of thermally annealed block copolymer films: (a) P3DOT, (b) P3DOT-b-PEG-10, (c) P3DOT-b-PEG-18, (d) P3DOT-b-PEG-40, (e) P3DOT-b-PEG-80, (f) P3DOT-b-PLLA-34, (g) P3DOT-b-PLLA-65

## 7.3 Out-of-plane Line-cut of block copolymer films

**Figure S48** Out-of-plane Line-cut of block copolymer films: (a) P3DOT, (b) P3DOT-b-PEG-10, (c) P3DOT-b-PEG-18, (d) P3DOT-b-PEG-40, (e) P3DOT-b-PEG-80, (f) P3DOT-b-PLLA-34, (g) P3DOT-b-PLLA-65

## 7.4 In-plane Line-cut of block copolymer films

**Figure S49** In-plane Line-cut of block copolymer films

## 7.5 Summary of fitted lamellar and π-π stacking distance

**Table S4** Summary of fitted lamellar and π-π stacking distance of all block copolymers

# Section S8 Differential Scanning Calorimetry

**Figure S50** Differential Scanning Calorigrams of all polymer samples

# Section S9 Inductively Coupled Plasma Mass Spectrometry Residual Pd Measurement (ICP-MS)

4.0 mg of each polymer was subjected to microwave‑assisted acid digestion in 4 mL concentrated HNO₃ at 100–110 °C for 2 h. 0.2 mL of the digested polymer acidic solution was diluted to a total volume of 10 mL with deionised water. ICP-MS was conducted with NexION 2000 ICP Mass Spectrometer to measure the residual Pd content in the polymer samples following scavenging.

| **Polymer** | **Pd / ppm** |
| --- | --- |
| P3DOT H/CHO | 3.77 |

# Section S10 Stability Measurement

**Figure S51** Operational stability performance of P3DOT-based polymers for 2000 seconds with voltage pulsing of 10 seconds. (*V*_DS_ = - 0.6V and *V*_GS_ = 0 to - 0.6 V).

# Section S11 References

S1. J. Liu, and R. D. McCullough, “End Group Modification of Regioregular Polythiophene through Postpolymerization Functionalization,” *Macromolecules* *35*, no. 27 (2002): 9882–9889, https://doi.org/10.1021/ma021362p.
